# Traditional Uses, Chemistry, Pharmacology, Toxicology and Quality Control of *Alhagi sparsifolia* Shap.: A Review

**DOI:** 10.3389/fphar.2021.761811

**Published:** 2021-10-14

**Authors:** Feng Wei, Xinzhou Yang, Kejian Pang, Hui Tang

**Affiliations:** ^1^ Key Laboratory of Xinjiang Phytomedicine Resource and Utilization, Ministry of Education, Pharmacy School of Shihezi University, Xinjiang, China; ^2^ School of Pharmaceutical Sciences, South-Central University for Nationalities, Wuhan, China

**Keywords:** alhagi sparsifolia shap, traditional uses, phytochemistry, pharmacology, quality control, toxicology

## Abstract

*Alhagi sparsifolia* Shap. (Kokyantak) is a ethnic medicine used in the Uyghur traditional medicine system for the treatment of colds, rheumatic pains, diarrhea, stomach pains, headaches, and toothaches, in addition to being an important local source of nectar and high-quality forage grass, and playing a crucial role in improving the ecological environment. Currently, approximately 178 chemical constituents have been identified from *A. sparsifolia*, including flavonoids, alkaloids, phenolic acids, and 19 polysaccharides. Pharmacological studies have already confirmed that *A. sparsifolia* has antioxidant, anti-tumor, anti-neuroinflammatory effects, hepatoprotective effects, renoprotective effects and immune regulation. Toxicological tests and quality control studies reveal the safety and nontoxicity of *A. sparsifolia*. Therefore, this paper systematically summarizes the traditional uses, botany, phytochemistry, pharmacology, quality control and toxicology of *A. sparsifolia*, in order to provide a beneficial reference of its further research.

## Introduction

The Uyghur system of medicine shares its source with ancient Greek-Arab medicine, one of the three traditional medicines in the world and dating back to more than 2,500 years in Xinjiang, China. The Uyghur system has been widely used in a clinical setting and is based on unique clinical theories. It continues to play an important and non-negligible role in preventing and curing diseases and maintaining public health. Uyghur medicine originated in the Western Regions during the ancient Neolithic period in Hotan (known as Yutian in ancient times) (Maituoheti et al., 2017). Ancient Uyghur physicians believed in Shamanism. They engaged in divination and demon removal and also used prayer and medicine to cure diseases, which formed the prototype of Uyghur medicine. Around the 5th century BC, the ancient ancestors of Uyghurs had advanced surgical techniques and methods of bone grafting (State Administration of Traditional Chinese Herbal Editorial Board, 2005). With the opening of the Silk Road and the deepening of cultural exchanges between China and the West, the Uyghurs absorbed the essence of traditional Chinese, Arabic, Persian, and Indian medicine to establish the Uyghur system of medicine, which had unique characteristics (Wang, 1994; Liu et al., 2016; Zhang, 2018). The humoral theory is the core of the theory of Uyghur medicine, which is gradually formed on the basis of the four major material theories and temperament theory (Kalbinur and Zhang, 2021). Uyghur medical humoral theory believes that hilits (humors) are produced on the basis of four major substances, namely, fire, air, water, and soil, and the four mijazs (temperamental qualities) namely, dry, hot, wet, and cold (Guan and Zhu, 1995; Aili, 1998a; Aili, 1998b). Different humors have different mijazs (Hamulati, 2003; Liu J. B. et al., 2014). The four mijazs, Sapra (bilious humor), kan (blood humor), belhem (mucus humor), sawda (black bile humor) coordinate with each other, maintaining a state of relative dynamic equilibrium to achieve normal physiological function and good health. Uyghur drugs are the “life code” for Uyghurs for longevity (Wang and Jiahan, 2011). Based on the natural temperament of people, Uyghur medicine classifies medicines from plant, animal, and mineral origins into eight medicinal properties, namely, wet, hot, dry, cold, wet-hot, wet-cold, dry-hot, and dry-cold; and nine medicinal tastes, namely, pungent, sweet, bitter, light, hot, sour, salty, astringent, strong, and oily. A combination pattern of medicinal properties-medicinal flavors-organ properties was established and the laws of dispensing Uyghur medicine prescriptions were elaborated (He, 2016). Uyghur medicine is trusted and affirmed by patients because it utilizes unique botanical drugs and formulas and is associated with a rapid onset of action and efficacy.

**FIGURE 1:**
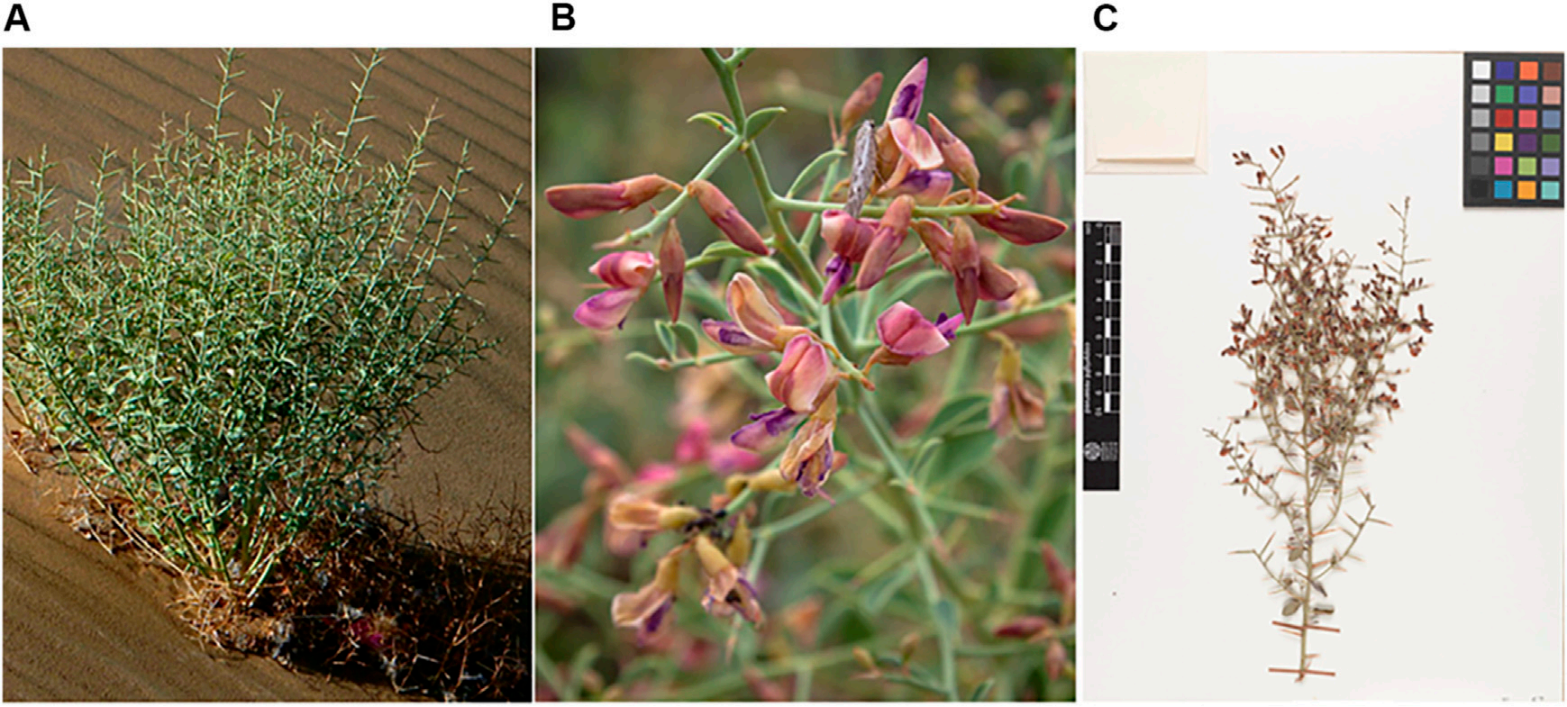
The figure shows the habitat **(A)**, flowers **(B)** and plant specimen **(C)** of *A. sparsifolia* (**(A)**: http://ppbc.iplant.cn/tu/5783326, **(B)**: https://www.inaturalist.org/photos/43301374, **(C)**: http://data.rbge.org.uk/herb/E00364493).

*A. sparsifolia* (syn. *A. kirghizorum* var. *sparsifolia* Shap. and *A. maurorum* subsp. *sparsifolium* (Shap.) Yakovlev) (Figure 1), belonging to the Fabaceae family is one such plant that is widely used in China (Inaturalist, 2021; Plant Photo Bank of China, 2021; Royal Botanic Garden Edinburgh, 2021; The Plant List, 2021). It is a typical species in arid and semi-arid desert regions and an important source of nectar and high-quality forage grass in the Tarim and Turpan basins (Sun, 1989; Ma, 1993). *A. sparsifolia* is mainly used in traditional Uyghur medicine to alleviate physical fatigue and treat colds, rheumatic pains, diarrhea, stomach pain, headaches, and toothaches and is called *Kokyantak* in the Uyghur language (Li et al., 1996). It is currently included in the Standard of Uyghur Medicinal Materials in the Xinjiang Autonomous Region (Xinjiang Medical Products Administration, 2010). The sugary secretion from its stem and leaves constitutes an important ethnomedicine called Tarangabin, which is effective in the treatment of abdominal pain, diarrhea, and dysentery. It has been included in the “Pharmaceutical Standards of the Ministry of Health of the People’s Republic of China” (Uyghur Medicines) (Chinese Pharmacopoeia Commission, 1998).

To date, 178 chemical constituents, including flavonoids, alkaloids, and phenolic acids, and 19 polysaccharide fragments have been identified from *A. sparsifolia*. Modern pharmacological studies reveal that the isolated components and crude extracts exhibit varied pharmacological activities including antioxidant, antitumor, anti-neuroinflammatory, hepatoprotective, and renoprotective effects. However, sufficient links between these pharmacological activities and the traditional uses of *A. sparsifolia* have not yet been established. Moreover, the bioactivities of only a few monomers have been studied. Besides, although its long-term efficacy has been demonstrated in its use as ethnic medicine, comprehensive reviews with respect to its safety and quality control are lacking. Therefore, in this review, we have summarized and analyzed, for the first time, existing studies (from 1985 to 2021) on the botany, traditional uses, phytochemistry, pharmacology, quality control, and toxicology of *A. sparsifolia*. Our review indicates that the research prospect for *A. sparsifolia* is very broad and worthy of further investigation.

## Material and Methods

The available information on *A. sparsifolia* was collected from scientific databases and cover from 1985 up to 2021. Information on *A. sparsifolia* was obtained from published materials, including monographs on medicinal plants, ancient and modern recorded classics, pharmacopoeias, Standard of Uyghur Medicinal Materials in Xinjiang Uyghur Autonomous region of China and electronic databases, such as Web of Science, Science Direct, Springer, Scifinder, X-MOL, PubMed, CNKI, Wanfang DATA, Google Scholar, Baidu Scholar, Flora of China (FOR). The search terms used for this review included “*Alhagi sparsifolia* Shap.“, “*A. kirghizorum* var. *sparsifolia Shap.*,” and “*A. maurorum* subsp. *sparsifolium* (Shap.) Yakovlev” all of which are accepted names and synonyms, “Saccharum alhagi*,”* “botanical characterization,” “flavonoid compounds,” “ethnomedicinal uses,” “quality standard,” “pharmacology,” and “toxicology.” Language restrictions were not applied during the search.

## Botanical Description, Geographic Distribution, and Taxonomy

### Botanical Description

*A. sparsifolia* is a deciduous shrub unique to the arid desert region of Xinjiang and is one of the “three treasures” of the Gobi Desert in preventing land desertification, resisting wind and sand erosion, and improving saline soil (Liu, 1985; Zhang et al., 2002; Arndt et al., 2004; Thomas et al., 2008; Jiang, 2017). There are seven species of the *Alhagi* genus worldwide, of which three are distributed in China and one is *A. sparsifolia* in the Flora of China (Zhang et al., 2008; Jin et al., 2014). In regions of high temperatures and low precipitation, the injured stems and leaves of *A. sparsifolia* secrete Tarangabin (Li et al., 1996; Wang, 2003; Mikeremu et al., 2016).

*A. sparsifolia* is a semi-shrub approximately 25–40 cm tall with an upright, glabrous stem having thin stripes. The leaves are alternate and ovate, obovate, or rounded ovoid, measuring approximately 8–15 mm long and 5–10 mm wide. The apex is round with short, hard tips, and the base is cuneate, entire, and glabrous with a short petiole. The flower is racemose, axillary, and 8–10 mm long. The rachis transforms into hard, sharp thorns that are 2–3 times as long as the leaves. The thorns of annual branches have 3–6 (or 3–8) flowers, but the older ones do not. The bract is subulate and up to 1 mm long, whereas the pedicel is 1–3-mm long. The calyx is campanulate, 4–5-mm long, and pubescent. The calyx teeth are triangular or subulate-triangular and one-third to one-fourth the length of the calyx tube. The corolla is reddish purple with a standard oblong-ovate shape and is 8–9-mm long, with an obtuse or truncated apex. The base is cuneate with a short petiole, the wing is oblong and three-quarters the length of the standard, and the carina is about the same length as the standard. The ovary and legume are linear and almost glabrous (Flora of China Editorial Committee, 1998). The harvest time of the various medicinal parts of *A. sparsifolia* differ. Flowers and leaves are collected in early summer (april to early May), Tarangabin is collected during midsummer (June to August), the seeds are collected in autumn (July to September), and the whole grass or aboveground parts are picked during the growing period of the year (Wang, 2003).

### Geographic Distribution

**FIGURE 2:**
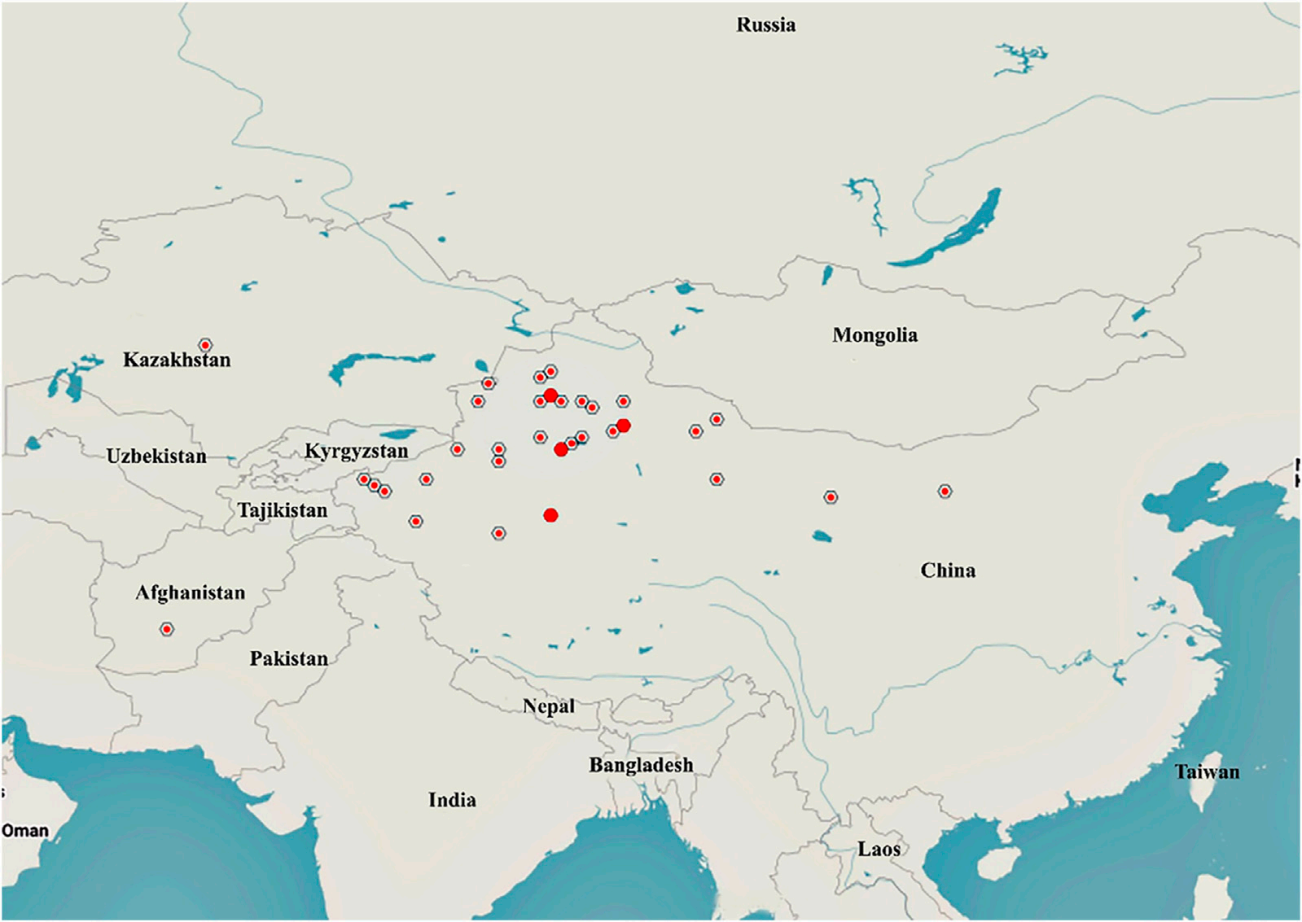
Distribution of *A. sparsifolia* in different countries and regions (https://www.gbif.org/species/2945088).

*A. sparsifolia* is distributed in Central and East Asia, mainly in China, Kazakhstan, Uzbekistan, Turkmenistan, Kyrgyzstan, and Tajikistan (GBIF, 2021; Nishanbaev et al., 2016) (Figure 2). The official website, Flora of China, states that *A. sparsifolia* plants are mainly found in Inner Mongolia, Gansu and Qinghai provinces, Xinjiang Autonomous Region, China (Flora of China Editorial Committee, 1998). The latest MaxEnt model simulations predict that the suitable habitats of *A. sparsifolia* will decrease due to the climate change scenarios of RCP 2.6 and 8.5 on the whole, indicating that the abundance of this species will show a downward trend in the future (Yang et al., 2017).

### Taxonomy

*A. sparsifolia* belongs to the Fabaceae family, which consists of over 24,505 species belonging to 946 genera, including *Glycine*, *Glycyrrhiza*, *Desmanthus*, *Lupinus*, *Medicago*, *Ormosia*, and *Styphnolobium*. Among them, the genus *Alhagi* includes eight species, namely, *A. graecorum* Boiss., *A. canescens* (Regel) B. Keller & Shap., *A. kirghisorum* Schrenk, *A. maurorum* Medik*., A. nepalensis* (D.Don) Shap., *A. pseudalhagi* (M. Bieb.) Desv. ex B. Keller & Shap., *A. sparsifolia* Shap., *A. sparsifolium* (Shap.) Shap. (http://www.worldfloraonline.org/taxon/wfo-0000198672/).

## Traditional Uses

*A. sparsifolia* was first found to be reported in “*Bei shi”* (AD 659) as *Yang ci*, whereas its medicinal value was first recorded in Tang Dynasty’s medical book, “*Ben Cao Shi Yi*” (AD 741) as *Cao mi*. In Ming Dynasty’s medical book, Compendium of Materia Medica (AD 1590), *A. sparsifolia* is listed as a “top grade” drug. It is a sweet, sour, and nontoxic drug usually used to treat abdominal pain, diarrhea, and dysentery. *A. sparsifolia* can be considered as “multiple medicinal parts in one plant,” i.e., different medicinal parts of the same plant have different pharmacological effects owing to differences in the chemical constituents and accumulation of the main components. Apart from being used routinely to treat colds and pains in various parts of the body, its leaves alleviate swelling and pain in joints; its flowers clear heat and detoxify the body; the whole plant alleviates colds and fevers, damp fever, and enteritis; and its seeds alleviate febrile dysentery and toothache (Yuan et al., 2012). Additionally, the therapeutic effects of Tarangabin depend on the route of administration. It is administered orally to treat hemorrhoids, as nasal drops to treat intractable headaches, and as eye drops to treat keratitis (Quan and Xu, 2009). As a traditional Uyghur medicine, *A. sparsifolia* is widely used in Uyghur medical practice in compound prescriptions with other botanical drugs. The prescription name, main composition, formulation, traditional and clinical uses, and prescription sources of *A. sparsifolia* are described in Table 1 (Chinese Medical Encyclopedia Committee, 2005).

**TABLE 1:** The traditional use of *A. sparsifolia* compound prescription in China.

Prescription name	Main composition	Extracts, formulations, usage, dosage	Traditional and clinical uses	Prescription sources
Maitibuhe Heiyari Xianbaier Tang	Tarangabin (45 g)/*Alhagi sparsifolia* Shap.*, Fructus Cassiae Fistulae* (45 g)/*Cassia fistula* L.*, Fructus Cordiae Dichotomae* (25 pcs)/*Cordia dichotoma* G.Forst.*, Fructus Ziziphi Jujubae* (25 pcs)/*Ziziphus jujuba* Mill.*, Semen Althaeae Roseae* (10 g)/*Alcea rosea* L.*, Herba Violae Tianshanicae* (10 g)/*Viola thianschanica* Maxim.*, Herba Chamomillae* (6 g)/*Chamaemelum nobile* (L.) All	Aqueous/Decoction/oral administratio/bid/100–200 ml each time	Treatment of conjunctival ophthalmia, ocular rim infection, eye pain, constipation	Yi Xue Zhi Mu Di (AD 1737)
Maitibuhe Aifeitimeng Tang	Tarangabin (120 g)*/Alhagi sparsifolia* Shap.*, Cortex Terminaliae Citrinae* (45 g)/*Terminalia citrina* (Gaertn.) Roxb.*, Cortex Terminaliae Billericae* (45 g)/*Terminalia bellirica* (Gaertn.) Roxb.*, Herba Dracocephali Moldavicae* (45 g)/*Dracocephalum moldavica* L.*, Fructus Terminaliae chebulae* (15 g)/*Terminalia chebula* Retz.*, Fructus Phyllanthi* (15 g)/*Phyllanthus emblica* L.*, Flos Lavandulae* (15 g)/*Lavandula angustifolia* Mill.*, Radix Valerianae* (15 g)/*Valeriana officinalis* L.*, Herba Anchusae* (30 g)/*Anchusa azurea Mill., Fructus Cassiae Fistulae* (30 g))/*Cassia fistula* L.*, Semen Cuscutae* (90 g)/*Cuscuta chinensis* Lam.*, Fos Nelumbinis* (12 g)/*Nelumbo nucifera* Gaertn.*, Semen Amygdalae* (120 g)/*Prunus amygdalus* Batsch*, Fructus Mume* (120 g)/*Prunus mume* (Siebold) Siebold & Zucc.*, Fructus Caryophylli* (6 g)/*Syzygium aromaticum* (L.) Merr. & L.M.Perry, *Cortex Cinnamomi* (6 g)/*Cinnamomum tamala* (Buch.-Ham.) T.Nees & Eberm.*, Herba Fumariae* (3 g)/*Fumaria officinalis* L.*, Rhizoma Polypodiodis* (12 g)*/Polypodiode snipponica* (Mett.) Ching, *Nipponicae* (12 g)/*Dioscorea nipponica* Makino*, Usnea* (9 g)/*Usnea diffracta* Vain.*, Semen Alpiniae Katsumadai* (9 g)/*Alpinia katsumadai* Hayata	Aqueous/Decoction/oral administratio/bid/124 ml each time	Treatment of insomnia, pain and retentionofurine	Hui Yao Fang (AD 1619)
Maizhuni Binaifeixie Migao	Tarangabin (150 g)*/Alhagi sparsifolia* Shap.*, Folium Sennae* (150 g)/*Senna alexandrina* Mill.*, Turpeth* (16 g)/*Operculina turpethum* (L.) Silva Manso*, Herba Anchusae* (16 g)/*Anchusa azurea Mill., Flos Rosae Rugosae* (16 g)/*Rosa rugosa* Thunb.*, Flos Violae Tianshanicae* (3 g)/*Viola thianschanica* Maxim.*, Fos Nelumbinis* (3 g)/*Nelumbo nucifera* Gaertn.*, Fructus Vitis Viniferae* (3 g)/*Vitis vinifera* L	Refined Honey/Honey Paste/oral administratio/qd/10 g each time	Treatment of febrile headache, eye pain, ear pain, conjunctival congestion, dizziness and constipation	Yi Xue Zhi Mu Di (AD 1737)
Maitibuhe Ainaluo Tang	Tarangabin (50 g)*/Alhagi sparsifolia* Shap.*, Fructus Mume* (100 g)/*Prunus mume* (Siebold) Siebold & Zucc.*, Semen Cichorii* (10 g)/*Cichorium intybus* L.*, Fructus Ziziphi Jujubae* (10 g)/*Ziziphus jujuba* Mill.*, Flos Rosae Rugosae* (13 g)/*Rosa rugosa* Thunb.*, Fructus Cordiae Dichotomae* (16 g)/*Cordia dichotoma* G.Forst.*, Flos Violae Tianshanicae* (16 g)/*Viola thianschanica* Maxim.*, Folium Sennae* (20 g)/*Senna alexandrina* Mill.*, Fructus Tamarindi Indicae* (31 g)/*Tamarindus indica* L.*, Fructus Cassiae Fistulae* (50 g))/*Cassia fistula* L	Aqueous/Decoction/oral administratio/bid/30–50 g each time	Treatment of headache, migraine, hematogenous dizziness, heartburn and thirst, typhoid fever, hepatomegaly	Bai Se Gong Dian (AD 1200)
Nukuyi Ailile Jinpaoye I	Tarangabin (50 g)*/Alhagi sparsifolia* Shap.*, Flos Violae Tianshanicae* (10 g)/*Viola thianschanica* Maxim.*, Semen Cichorii* (10 g)/*Cichorium intybus* L.*, Cortex Terminaliae citrinae* (31 g)/*Terminalia citrina* (Gaertn.) Roxb	Aqueous/Decoction/oral administratio/bid-tid/30–60 ml each time	Treatment of febrile headache	Yi Xue Zhi Mu Di (AD 1737)
	*Fructus Cassiae Fistulae* (31 g))/*Cassia fistula* L.*, Fructus Ziziphi Jujubae* (31 g)/*Ziziphus jujuba* Mill.*, Fructus Cordiae Dichotomae* (56 g)/*Cordia dichotoma* G.Forst.*, Fructus Tamarindi Indicae* (60 g)/*Tamarindus indica* L.*, Fructus Mume* (100 g)/*Prunus mume* (Siebold) Siebold & Zucc			
Nukuyi Ailile Jinpaoye Ⅱ	Tarangabin (150 g)*/Alhagi sparsifolia* Shap.*, Cortex Terminaliae citrinae* (30 g)/*Terminalia citrina* (Gaertn.) Roxb.*, Fructus Mume* (30 pcs)/*Prunus mume* (Siebold) Siebold & Zucc.*, Fructus Cordiae Dichotomae* (30 pcs)/*Cordia dichotoma* G.Forst.*, Fructus Ziziphi Jujubae* (30 pcs)/*Ziziphus jujuba* Mill.*, Fructus Tamarindi Indicae* (60 pcs)/*Tamarindus indica* L.*, Flos Violae Tianshanicae* (9 g)/*Viola thianschanica* Maxim.*, Semen Cichorii*(9 g)/*Cichorium intybus* L.*, Fructus Cassiae Fistulae* (30 g))/*Cassia fistula* L	Aqueous/Decoction/oral administratio/bid/100 ml each time	Treatment of febrile headache, migraine, fever and thirst	A Ri Fu Yan Fang (AD 1556–1,662)
Nukuyi Pawake Jinpaoye	Tarangabin (60 g)*/Alhagi sparsifolia* Shap.*, Fructus Mume* (30 pcs)/*Prunus mume* (Siebold) Siebold & Zucc.*, Fructus Ziziphi Jujubae* (30 pcs)/*Ziziphus jujuba* Mill.*, Fructus Cordiae Dichotomae* (30 pcs)/*Cordia dichotoma* G.Forst.*, Fructus Tamarindi Indicae* (30 g)/*Tamarindus indica* L	Aqueous/Decoction/oral administratio/bid/50–100 ml each time	Treatment of fever, meningitis, migraine	Yi Xue Zhi Mu Di (AD 1737)
Mengziji Saiweida Chengshuji	Tarangabin (70 g)*/Alhagi sparsifolia* Shap.*, Herba Anchusae* (25 g)/*Anchusa azurea Mill., Rhizoma Polypodiodis* (25 g)*/Polypodiode snipponica* (Mett.) Ching, *Nipponicae* (25 g)/*Dioscorea nipponica* Makino*, Fructus Cordiae Dichotomae* (25 g)/*Cordia dichotoma* G.Forst.*, Flos Lavandulae* (25 g)/*Lavandula angustifolia* Mill.*, Cortex Terminaliae chebulae* (16 g)/*Terminalia chebula* Retz.*, Herba Hyssopi* (16 g)/*Hyssopus officinalis* L.*, Flos Violae Tianshanicae* (16 g)/*Viola thianschanica* Maxim	Aqueous/Decoction oral administratio/bid-tid/50 ml each time	Treatment of meningitis	Bai Di Yi Yao Shu (AD 1368)
Maizhuni Binaifeixie Migao I	Tarangabin (60 g)*/Alhagi sparsifolia* Shap.*, Flos Violae Tianshanicae* (30 g)/*Viola thianschanica* Maxim.*, Semen Amygdalae* (30 g)/*Prunus amygdalus* Batsch*, Mastix* (15 g)/*Pistacia lentiscus* L.*, Radix et Rhizoma Glycyrrhizae* (15 g)/*Glycyrrhiza uralensis* Fisch. ex DC.*, Turpeth* (60 g)/*Operculina turpethum* (L.) Silva Manso*, Fructus Cassiae Fistulae* (60 g))/*Cassia fistula* L	Aqueous & Sugar/Honey Paste/oral administratio/bid/5 g each time (adults); bid/3 g each time (children)	Treatment of cough, intestinal obstruction, phlegm, gastritis	A Ri Fu Yan Fang (AD 1556–1,662)
Aibi Taipi Xiaowan	Tarangabin (3 g)*/Alhagi sparsifolia* Shap.*, Papaveris Pericarpium* (3 g)/*Papaver somniferum* L.*, Rmmi Rabicum* (3 g)/*Senegalia senegal* (L.) Britton*, Gummi Tragacanthae* (3 g)/*Astragalus gummifer* Labill.*, Radix et Rhizoma Glycyrrhizae* (3 g)/*Glycyrrhiza uralensis* Fisch. ex DC.*, Semen Lagenariae Sicerariae* (3 g)/*Lagenaria siceraria* (Molina) Standl.*, Semen Amygdalae* (3 g)/*Prunus amygdalus* Batsch*, Semen Cucumeris* (3 g)/*Cucumis satiuus* L	Pill/oral administratio/bid/1 pcs each time	Treatment of habitual typhoid fever, tuberculosis, back pain, cough and phlegm	Bai Di Yi Yao Shu (AD 1368)
		Pill		
Xieribiti Ounabi Tangjiang	Tarangabin (300 g)*/Alhagi sparsifolia* Shap.*, Fructus Mume* (60 g)/*Prunus mume* (Siebold) Siebold & Zucc.*, Fructus Ziziphi Jujubae* (30 g)/*Ziziphus jujuba* Mill.*, Fructus Tamarindi Indicae* (100 g)/*Tamarindus indica* L.*, Flos Violae Tianshanicae* (60 g)/*Viola thianschanica* Maxim.*, Turpeth* (60 g)/*Operculina turpethum* (L.) Silva Manso, *Resina Scammoniae* (3 g)/*Convovulus scammonia* L., *Stigma Croci* (1 g)/*Crocus sativus* L	Aqueous/Syrup/oral administratio/tid/50 ml each time	Treatment of hyperthermic typhoid fever arising from the excessive influence of hot body humor such as bilious or blood	A Ri Fu Yan Fang (AD 1556–1,662)
Xieribiti Kushuxi Tangjiang	Tarangabin (100 g)*/Alhagi sparsifolia* Shap.*, Semen Cichorii* (15 g)/*Cichorium intybus* L.*, Herba Moslae* (15 g)/*Mosla chinensis* Maxim.*, Semen Cuscutae* (20 g)/*Cuscuta chinensis* Lam.*, Radix Foeniculi* (35 g)/*Foeniculum vulgare* Mill.*, Radix et Rhizoma Glycyrrhizae* (50 g)/*Glycyrrhiza uralensis* Fisch. ex DC.*, Radix Cichorii* (30 g)/*Cichorium intybus* L.*, Semen Cuscutae* (30 g)/*Cuscuta chinensis* Lam.*, Semen Cucumeris* (30 g)/*Cucumis satiuus* L	Aqueous & Sugar/Syrup/oral administratio/tid/100 ml each time	Treatment of respiratory system diseases and heart, liver and gastrointestinal diseases, fever and cough, complicated typhoid fever, febrile heart and liver deficiency, unfavorable urination and poor bowel movement	Yi Xue Zhi Mu Di (AD 1737)
Maitibuhe Aifeisanting Tang	Tarangabin (30 g)*/Alhagi sparsifolia* Shap.*, Herba Absinthii* (15 g)/*artemisia absinthium* L.*, Flos Rosae Rugosae* (20 g)/*Rosa rugosa* Thunb.*, Fructus Tamarindi Indicae* (60 g)/*Tamarindus indica* L	Aqueous/Decoction/oral administratio/tid/100 ml each time	Treatment digestive disorders, such as spleen and stomach diseases, hyperthermic typhoid fever, fever and headache, indigestion	Zhu Yi Dian (AD 1040–1,050)
Maizhuni Binaifeixie Migao Ⅱ	Tarangabin (60 g)*/Alhagi sparsifolia* Shap.*, Flos Violae Tianshanicae* (30 g)/*Viola thianschanica* Maxim.*, Semen Amygdalae* (30 g)/*Prunus amygdalus* Batsch*, Mastix* (15 g)/*Pistacia lentiscus* L.*, Radix et Rhizoma Glycyrrhizae* (15 g)/*Glycyrrhiza uralensis* Fisch. ex DC.*, Turpeth* (60 g)/*Operculina turpethum* (L.) Silva Manso*, Fructus Cassiae Fistulae* (60 g)/*Cassia fistula* L	Aqueous & Sugar/Honey Paste/oral administratio/tid/5 g each time(adults); qd/1 g each time(children)	Treatment of intestinal and respiratory disorders, such as intestinal constipation and obstruction, abnormal bilious and mucinous increase, cough and phlegm	A Ri Fu Yan Fang (AD 1556–1,662)
Maitibuhe Mengziji Chengshuji Ⅲ	Tarangabin (30 g)*/Alhagi sparsifolia* Shap.*, Fructus Ziziphi Jujubae* (15 g)/*Ziziphus jujuba* Mill.*, Fructus Solani Nigri* (10 g)/*Solanum nigrum* L.*, Semen Rutae* (6 g)/*Ruta graveolens* L	Aqueous/Decoction/oral administratio/tid × 3 d	Treatment of increased abnormal body humor in upper extremity joints, upper extremity soreness	Bai Se Gong Dian (AD 1200)
Maitibuhe Surenjiang Tang Ⅱ	Tarangabin (60 g)*/Alhagi sparsifolia* Shap.*, Folium Sennae* (20 g)/*Senna alexandrina* Mill.*, Flos Rosae Rugosae* (12 g)/*Rosa rugosa* Thunb.*, Cortex Terminaliae citrinae* (12 g)/*Terminalia citrina* (Gaertn.) Roxb.*, Bulbus Colchici* (6 g)/*Colchicum autumnale* L.*, Radix Foeniculi* (6 g)/*Foeniculum vulgare* Mill.*, Herba Foeniculi* (6 g)/*Foeniculum vulgare* Mill.*, Fructus Apii* (6 g)/*Apium graveolens* L.*, Herba Centaurii* (6 g)/*Centaurium erythraea* Rafn*, Fructus Anethi* (6 g)/*Anethum graveolens* L.*, Herba Anchusae* (10 g)/*Anchusa azurea Mill., Herba Melissae Axillaris* (10 g)/*Melissa axillaris* (Benth.) Bakh.f	Aqueous/Decoction/oral administratio/tid/50–100 ml each time	Treatment of joint pain, urinary discomfort, poor bowel movement, arthritis and swelling	A Ri Fu Yan Fang (AD 1556–1,662)
Xieribiti Mengziji Maxire Tangjiang	Tarangabin (60 g)*/Alhagi sparsifolia* Shap.*, Flos Violae Tianshanicae* (16 g)/*Viola thianschanica* Maxim.*, Flos Nelumbinis* (16 g)/*Nelumbo nucifera* Gaertn.*, Flos Rosae Rugosae* (16 g)/*Rosa rugosa* Thunb.*, Semen Cichorii* (9 g)/*Cichorium intybus* L.*, Radix Cichorii* (18 g)/*Cichorium intybus* L.*, Fructus Ziziphi Jujubae* (7 pcs)/*Ziziphus jujuba* Mill	Aqueous/Syrup/oral administratio/bid/50 ml each time	Treatment of skin diseases such as febrile dermatitis, various inflammations and swellings inside and outside the body, and dry stools	Bao jian Yao Yuan (AD 1556–1,662)
Mengziji Bairese Chengshuji Ⅲ	Tarangabin (100 g)*/Alhagi sparsifolia* Shap.*, Fructus Anethi* (15 g)/*Anethum graveolens* L., *Fructus Apii* (15 g)/*Apium graveolens* L.*, Semen Nigellae* (15 g)/*Nigella glandulifera* Freyn & Sint.*, Folium Sennae* (15 g)/*Senna alexandrina* Mill.*, Radix Foeniculi* (30 g)/*Foeniculum vulgare* Mill.*, Rhizoma Zingiberis* (30 g)/*Zingiber officinale* Roscoe*, Flos Lavandulae* (30 g)/*Lavandula angustifolia* Mill.*, Fructus Caricae* (25 g)/*Ficus carica* L.*, Radix Apii* (25 g)/*Apium graveolens* L.*, Radix et Rhizoma Glycyrrhizae* (25 g)/*Glycyrrhiza uralensis* Fisch. ex DC.*, Radix et Rhizoma Nardostachyos* (10 g)/*Nardostachys jatamansi* (D.Don) DC.*, Fructus Vitis Viniferae* (150 g)/*Vitis vinifera* L	Aqueous/Decoction/oral administratio/tid/150 ml each time	Treatment of skin hypopigmentation diseases, such as vitiligo	Bai Di Yi Yao Shu (AD 1368)
Musili Bairese Qingchuji	Tarangabin (150 g)*/Alhagi sparsifolia* Shap.*, Cortex Terminaliae citrinae* (20 g)/*Terminalia citrina* (Gaertn.) Roxb.*, Fructus Terminaliae chebulae* (20 g)/*Terminalia chebula* Retz.*, Cortex Terminaliae billericae* (20 g)/*Terminalia bellirica* (Gaertn.) Roxb.*, Fructus Phyllanthi* (20 g)/*Phyllanthus emblica* L.*, Herba Anchusae* (15 g)/*Anchusa azurea Mill., Herba Melissae Axillaris* (15 g)/*Melissa axillaris* (Benth.) Bakh.f.*, Radix Foeniculi* (15 g)/*Foeniculum vulgare* Mill.*, Fructus Anethi* (15 g)/*Anethum graveolens* L., *Radix et Rhizoma Glycyrrhizae* (15 g)/*Glycyrrhiza uralensis* Fisch. ex DC.*, Fructus Solani Nigri* (15 g)/*Solanum nigrum* L.*, Semen Cuscutae* (30 g)/*Cuscuta chinensis* Lam.*, Folium Sennae* (30 g)/*Senna alexandrina* Mill.*, Radix Plumbinis* (30 g)/*Plumbago zeylanica* L.*, Fructus Ziziphi Jujubae* (30 g)/*Ziziphus jujuba* Mill.*, Fructus Cordiae Dichotomae* (10 g)/*Cordia dichotoma* G.Forst.*, Turpeth* (10 g)/*Operculina turpethum* (L.) Silva Manso	Aqueous/Decoction/oral administratio/bid/150 ml each time	Treatment of abnormal increased body humor, irregular bowel movements, bloating and edema, vitiligo	Bai Di Yi Yao Shu (AD 1368)
Xieribiti Ounabi Murekaibi Tangjiang	Tarangabin (60 g)*/Alhagi sparsifolia* Shap.*, Fructus Ziziphi Jujubae* (10 pcs)/*Ziziphus jujuba* Mill.*, Fructus Vitis Viniferae* (10 pcs)/*Vitis vinifera* L.*, Fructus Mume*(10 pcs)/*Prunus mume* (Siebold) Siebold & Zucc.*, Flos Nelumbinis* (10 g)/*Nelumbo nucifera* Gaertn.*, Semen Althaeae Roseae* (10 g)/*Alcea rosea* L.*, Fructus Solani Nigri* (10 g)/*Solanum nigrum* L.*, Folium Isatidis* (10 g)/*Isatis tinctoria* L.*, Herba Fumariae* (15 g)/*Fumaria officinalis* L.*, Folium Sennae* (15 g)/*Senna alexandrina* Mill.*, Semen Cucumeris* (15 g)/*Cucumis satiuus* L.*, Semen melo* (15 g)/*Cucumis melo* L.*, Semen Amygdalae* (15 g)/*Prunus amygdalus* Batsch*, Lignum Santali Albi* (6 g)/*Santalum album* L.*, Herba Swertiae* (6 g)/*Swertia diluta* (Turcz.) Benth. & Hook.f.*, Flos Rosae Rugosae* (6 g)/*Rosa rugosa* Thunb.*, Fructus Cassiae Fistulae* (30 g)/*Cassia fistula* L	Aqueous/Syrup/oral administratio/bid/60 ml each time	Treatment of gynecological diseases such as cervicitis, uterine sores, vulvar itching, uterine pain	Yi Xue Zhi Mu Di (AD 1737)

## Phytochemistry

**FIGURE 3:**
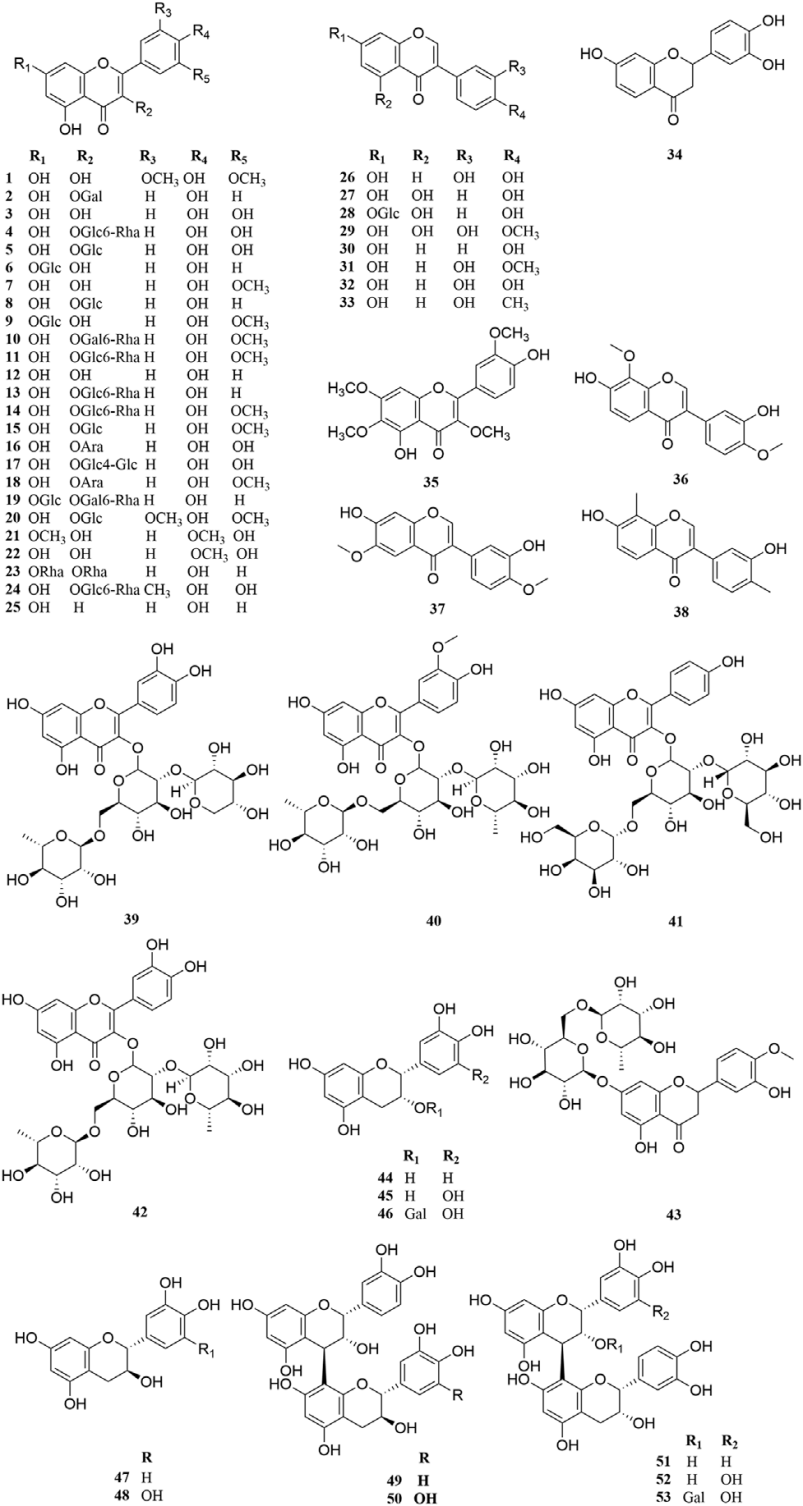
Chemical structures of flavonoids (**1–53**).

**FIGURE 4:**
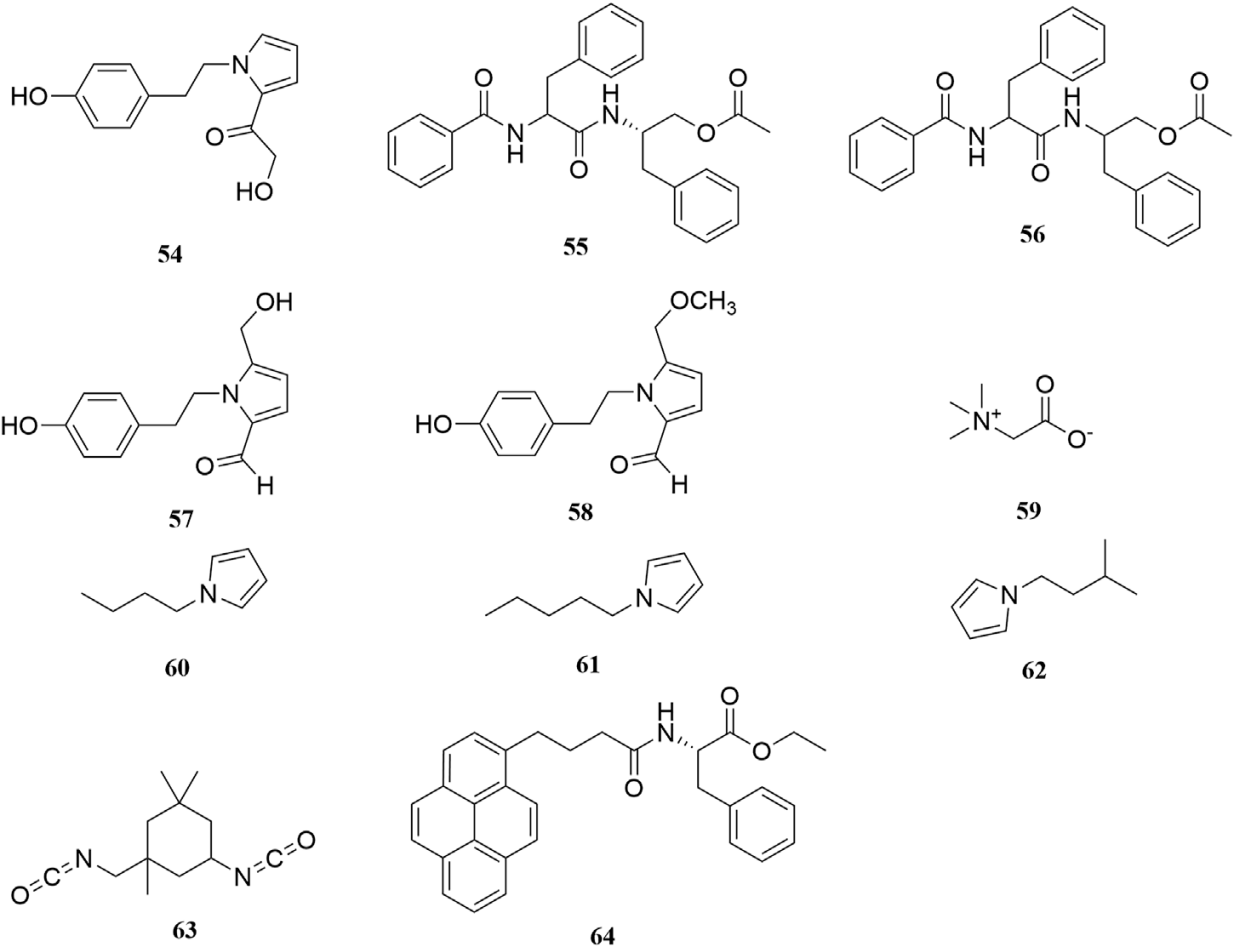
Chemical structures of alkaloids (**54–64**).

**FIGURE 5:**
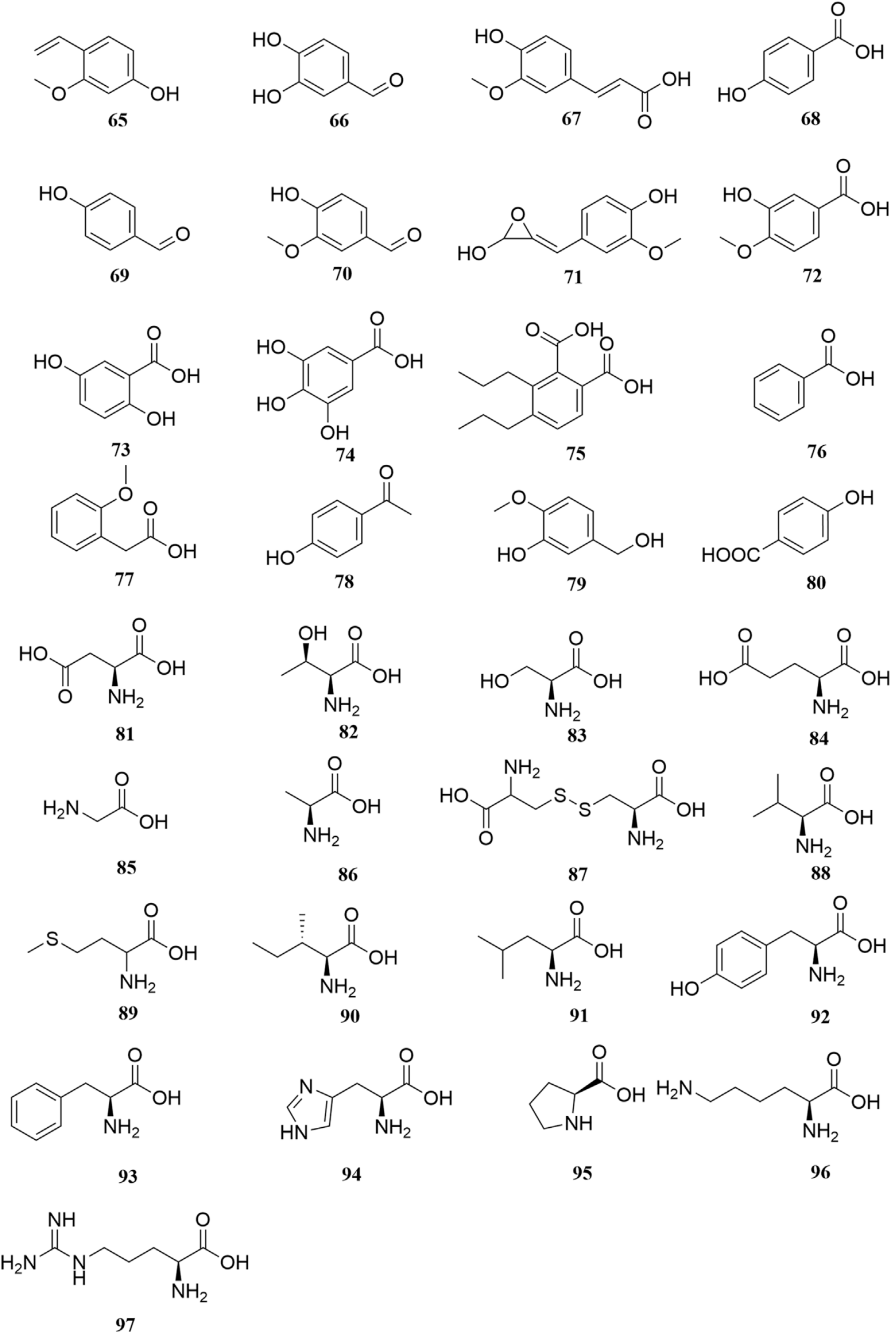
Chemical structures of phenols, phenolic acids and amino acids (**65–97**).

**FIGURE 6:**
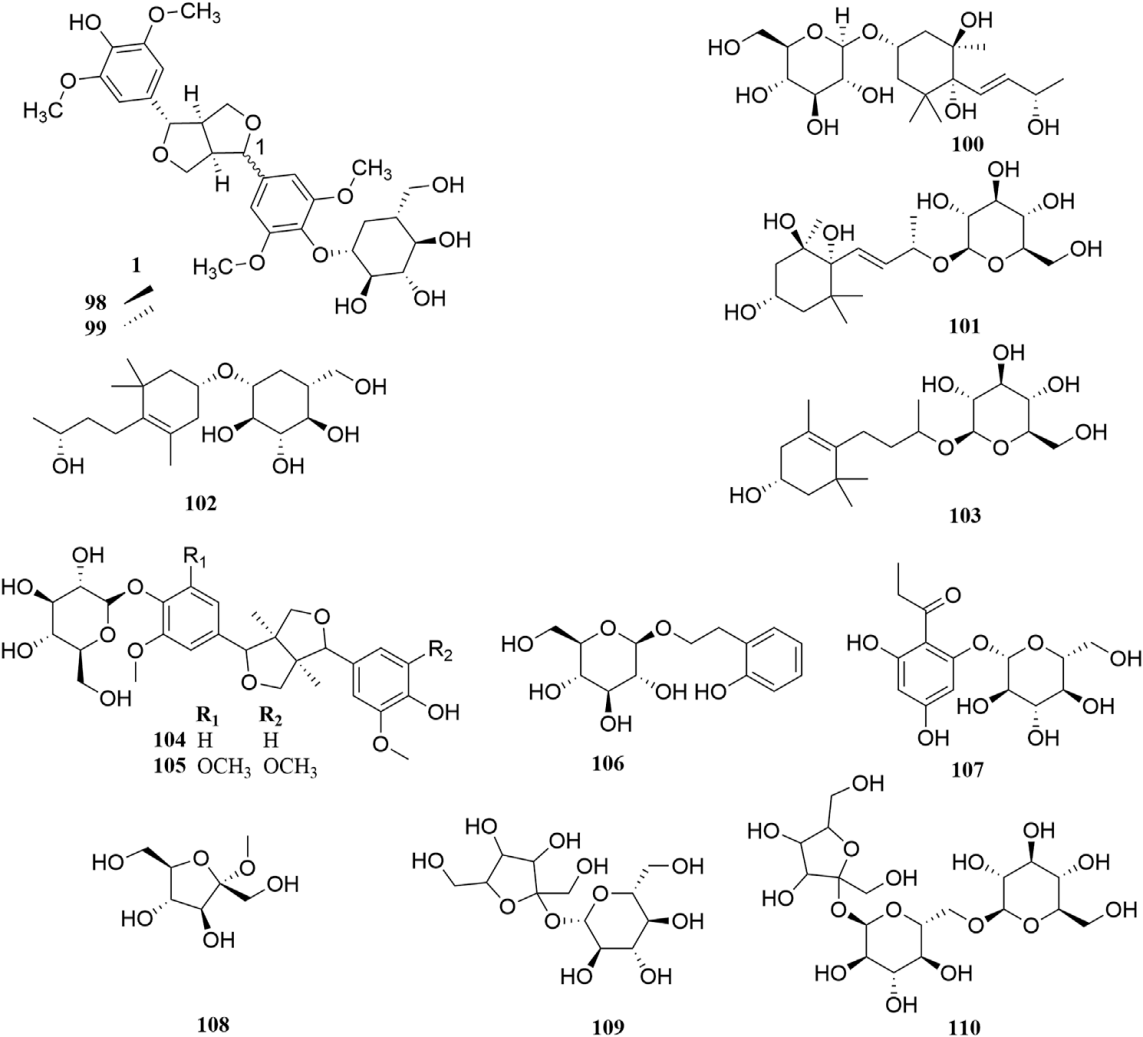
Chemical structures of saccharides and glycosides (**98–110**).

**FIGURE 7:**
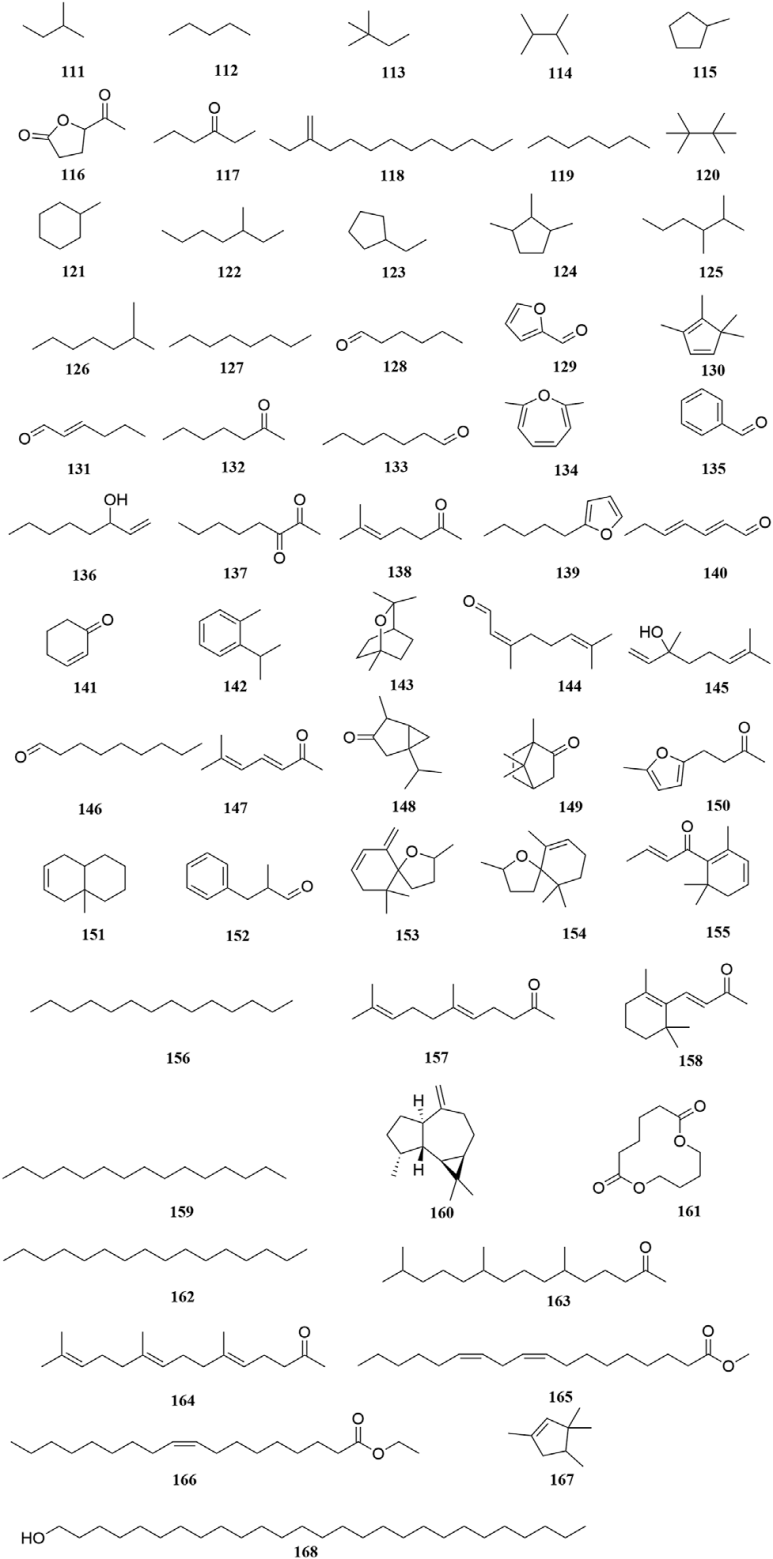
Chemical structures of volatile oils (**111–168**).

**FIGURE 8:**
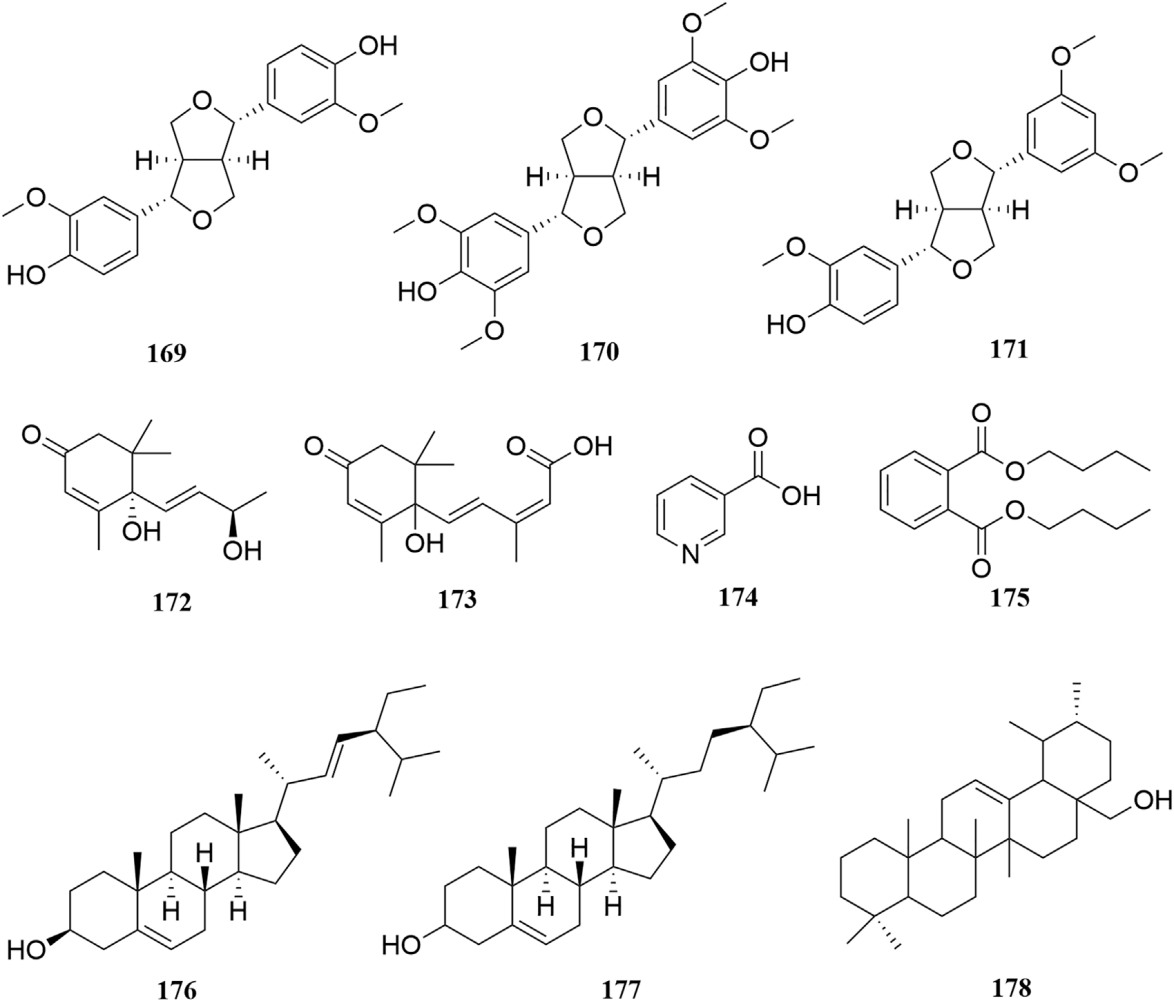
Chemical structures of other constituents (**169–178**).

Investigation of chemical constituents from *A. sparsifolia* began in 1997. To date, approximately 178 chemical constituents have been identified, including flavonoids, alkaloids and phenolic acids, and 19 polysaccharides. Among them, flavonoids and polysaccharides are the predominant and characteristic constituents. The chemical constituents that have been identified are listed in Table 2 and their corresponding structures in Figures 3–8.

**TABLE 2:** Chemical components isolated and structurally identified from *A. sparsifolia* (MF = Molecular Formula).

No	Chemical constituents	MF	Extracts	Parts	References
	**Flavonoids**				
1	butin	C_15_H_12_O_5_	EtOAc	Aerial part	Ma et al. (2018a)
2	kaempferol-7-*O*-*β*-d-glucopyranoside	C_21_H_20_O_11_	EtOAc	Flowers	Guo et al. (2016)
3	isorhamnetin	C_16_H_12_O_7_	EtOAc	Flowers	Guo et al. (2016)
4	isorhamnetin-3-*O*-*β*-rutinoside	C_28_H_32_O_16_	BuOH	Stem	Ouyang et al. (2018)
5	kaempferol	C_15_H_10_O_6_	EtOAc	Flowers	Guo et al. (2016)
6	isoquercitrin	C_21_H_20_O_12_	EtOAc	Flowers	Guo et al. (2016)
7	syringetin	C_17_H_14_O_8_	EtOAc	Flowers	Guo et al. (2016)
8	kaempferol-3-*O*-*β*-d-galactopyranoside	C_21_H_20_O_11_	EtOAc	Flowers	Guo et al. (2016)
9	kaempferol-3-*O*-*β*-d-glucopyranoside	C_21_H_20_O_11_	EtOAc	Flowers	Guo et al. (2016)
10	isorhamnetin-7-*O*-*β*-d-glucopyranoside	C_22_H_22_O_12_	EtOAc	Flowers	Guo et al. (2016)
11	isorhamnetin-3-*O*-robinobioside	C_28_H_32_O_16_	BuOH	Stem	Ouyang et al. (2018)
12	quercetin	C_15_H_10_O_7_	EtOA	Flowers	Guo et al. (2016)
13	quercetin-3-*O*-rutinoside	C_27_H_30_O_16_	BuOH	Flowers	Muratova et al. (2019)
14	kaempferol-3-*O*-rutinoside	C_27_H_30_O_15_	BuOH	Flowers	Muratova et al. (2019)
15	quercetin-3-*O*-*β*-d-glucopyranoside	C_21_H_20_O_12_	EtOH	Aerial part	Zhou et al. (2017)
16	isorhamnetin-3-*O*-glucoside	C_22_H_22_O_12_	BuOH	Aerial part	Su et al. (2008)
17	guaijaverin	C_20_H_18_O_11_	EtOH	Secretory	Guo et al. (2020)
18	quercetin-3-*O*-maltoside	C_27_H_30_O_16_	EtOH	Secretory	Guo et al. (2020)
19	isorhamnetin-3-*O*-arabinoside	C_21_H_20_O_12_	EtOH	Secretory	Guo et al. (2020)
20	kaempferol-3-*O*-*α*-l-rhamnopyranosyl(1→6)-*β*-d-glucopyranosyl-7-*O*-*β*-d-glucopyranoside	C_33_H_40_O_19_	EtOH	Secretory	Guo et al. (2020)
21	syringetin-3-*O*-*β*-D-glucoside	C_23_H_24_O_13_	EtOH	Secretory	Guo et al. (2020)
22	ombuin	C_17_H_14_O_7_	EtOH	Secretory	Guo et al. (2020)
23	tamarixetin	C_16_H_12_O_7_	EtOH	Secretory	Guo et al. (2020)
24	kaempferitrin	C_27_H_30_O_14_	EtOH	Secretory	Guo et al. (2020)
25	3′-*O*-methylquercetin-3-*O*-*α*-rutinoside	C_28_H_32_O_16_	EtOH	Secretory	Guo et al. (2020)
26	apigenin	C_15_H_10_O_5_	EtOH	Secretory	Guo et al. (2020)
27	3′,4′,7-trihydroxyisoflavone	C_15_H_10_O_5_	BuOH	Aerial part	Liu et al. (2019a)
28	genistein	C_15_H_10_O_5_	EtOAc	Flowers	Guo et al. (2016)
29	genistin	C_21_H_20_O_10_	EtOAc	Flowers	Guo et al. (2016)
30	pratensein	C_16_H_12_O_6_	BuOH	Stem	Ouyang et al. (2018)
31	formonoetin	C_16_H_12_O_4_	EtOH	Aerial part	Zhou et al. (2017)
32	3′,7-dihydroxyl-4′-methoxylisoflavone	C_16_H_12_O_5_	EtOH	Aerial part	Zhou et al. (2017)
33	3′,4′,7-trihydroxylisoflavone	C_15_H_10_O_5_	EtOH	Aerial part	Zhou et al. (2017)
34	3′,7-dihydroxy-4′-methylisoflavone	C_16_H_12_O_4_	EtOH	Secretory	Guo et al. (2020)
35	chrysoplenol B	C_19_H_18_O_8_	BuOH	Flowers	Muratova et al. (2019)
36	3′,7-dihydroxyl-4′,8-dimethoxylisoflavone	C_17_H_14_O_6_	EtOH	Aerial part	Zhou et al. (2017)
37	3′,7-dihydroxyl-4′,6-dimethoxylisoflavone	C_17_H_14_O_6_	EtOH	Aerial part	Zhou et al. (2017)
38	3′,7-dihydroxy-4′,8-dimethylisoflavone	C_17_H_14_O_4_	EtOH	Secretory	Guo et al. (2020)
39	quercetin-3-*O*-(2-*β*-d-xylopyranosyl)-*β*-d-rutinoside	C_32_H_38_O_20_	EtOH	Aerial part	Zhou et al. (2017)
40	typhaneoside	C_34_H_42_O_20_	EtOH	Aerial part	Zhou et al. (2017)
41	kaempferol-3-*O*-*β*-d-glucosyl-(1→2)-*O*-[*α*-l-rhamnosyl(1→6)]-*β*-d-galactoside	C_33_H_40_O_21_	BuOH	Flowers	Muratova et al. (2019)
42	quercetin-3-*O*-(2″,6″-di-*O*-*α*-l-rhamnopyranosyl)-*β*-d-glucopyranoside	C_33_H_40_O_20_	EtOH	Aerial part	Zhou et al. (2017)
43	hesperidin	C_28_H_34_O_15_	EtOH	Secretory	Guo et al. (2020)
44	(-)-epicatechin	C_15_H_14_O_6_	EtOH	Epigeal	Malik et al. (1997)
45	(-)-epigallocatechin	C_15_H_14_O_7_	EtOH	Epigeal	Malik et al. (1997)
46	(-)-epigallocatechin-3-*O*-gallate	C_22_H_18_O_11_	EtOH	Epigeal	Malik et al. (1997)
47	(+)-catechin	C_15_H_14_O_6_	EtOH	Epigeal	Malik et al. (1997)
48	(+)-gallocatechin	C_15_H_14_O_7_	EtOH	Epigeal	Malik et al. (1997)
49	(-)-epigauocatechin-3-*O*-ganate-(4*β*-8)-(-)-epicateehin	C_30_H_26_O_12_	EtOH	Epigeal	Malik et al. (1997)
50	(-)-epicatechin-(4*β*-8)-(+)-gallocatechin	C_30_H_26_O_13_	EtOH	Epigeal	Malik et al. (1997)
51	proanthocyanidin B-2	C_30_H_26_O_12_	EtOH	Epigeal	Malik et al. (1997)
52	proanthoeyanidin B-1	C_30_H_26_O_13_	EtOH	Epigeal	Malik et al. (1997)
53	(-)-epigallocateehin-(4*β*-8)-(-)-epicatechin	C_37_H_30_O_17_	EtOH	Epigeal	Malik et al. (1997)
102	**Alkaloids**				
54	alhagifoline A	C_14_H_15_NO_3_	BuOH	Aerial part	Zou et al. (2012)
55	aurantiamide acetate	C_27_H_28_N_2_O_4_	EtOH	Aerial part	Zhou et al. (2017)
56	aurantiamide	C_27_H_28_N_2_O_4_	EtOH	Aerial part	Zhou et al. (2017)
57	pyrrolezanthine	C_14_H_15_NO_3_	BuOH	Aerial part	Zou et al. (2012)
58	pyrrolezanthine-6-methyl ether	C_15_H_17_NO_3_	BuOH	Aerial part	Zou et al. (2012)
59	betaine	C_5_H_11_NO_2_	Et_2_O	Aerial part	Liu G. C. et al. (2014)
60	1-butyl-1*H*-pyrrole	C_8_H_13_N	Aqueous	Secretory	Cheng, (2010)
61	1-pentyl-1*H*-pyrrole	C_9_H_15_N	Aqueous	Secretory	Cheng, (2010)
62	1-isoamylpyrrole	C_9_H_15_N	Aqueous	Secretory	Cheng, (2010)
63	5-isocyanato-1-(isocyanatomethyl)-1,3,3-trimethyl-cyclohexane	C_12_H_1_8N_2_O_2_	Aqueous	Secretory	Cheng, (2010)
64	ethyl ester	C_31_H_29_NO_3_	Aqueous	Secretory	Cheng, (2010)
102	**Phenols, carboxylic acids, phenolic acids and amino acids**				
65	3-methoxy-4-vinylphenol	C_9_H_10_O_2_	EtOAc	Aerial part	Ma et al. (2018b)
66	3,4-dihydroxybenzaldehyde	C_7_H_6_O_3_	BuOH	Aerial part	Ma et al. (2018b)
67	ferulic acid	C_10_H_10_O_4_	BuOH	Stem	Ouyang et al. (2018)
68	*p*-hydroxybenzoic acid	C_7_H_6_O_3_	BuOH	Stem	Ouyang et al. (2018)
69	*p*-hydroxybenzaldehyde	C_7_H_6_O_2_	BuOH	Stem	Ouyang et al. (2018)
70	vanillin	C_8_H_8_O_3_	BuOH	Stem	Ouyang et al. (2018)
71	epoxyconiferyl alcohol	C_10_H_10_O_4_	BuOH	Stem	Ouyang et al. (2018)
72	isovanillic acid	C_8_H_8_O_4_	EtOAc	Flowers	Guo et al. (2016)
73	gentisic acid	C_7_H_6_O_4_	EtOAc	Flowers	Guo et al. (2016)
74	gallic acid	C_7_H_6_O_5_	EtOAc	Flowers	Guo et al. (2016)
75	dipropylphthalate	C_14_H_18_O_4_	EtOAc	Secretory	Cheng et al. (2014)
76	benzoic acid	C_7_H_6_O_2_	BuOH	Flowers	Muratova et al. (2019)
77	methoxyphenyl acetic acid	C_9_H_10_O_3_	EtOH	Aerial part	Zhou et al. (2017)
78	4′-hydroxylacetophenone	C_8_H_8_O_2_	EtOH	Aerial part	Zhou et al. (2017)
79	3-hydroxyl-4-methoxybenzyl alcohol	C_8_H_10_O_3_	EtOH	Aerial part	Zhou et al. (2017)
80	4-hydroylphenoyl	C_7_H_6_O_3_	EtOH	Aerial part	Zhou et al. (2017)
81	aspartic acid	C_4_H_7_NO_4_	Et_2_O	Aerial part	Liu G. C. et al. (2014)
82	l-threonine	C_4_H_9_NO_3_	Et_2_O	Aerial part	Liu G. C. et al. (2014)
83	*β*-hydroxyalanine	C_3_H_7_NO_3_	Et_2_O	Aerial part	Liu G. C. et al. (2014)
84	glutamic acid	C_5_H_9_NO_4_	Et_2_O	Aerial part	Liu G. C. et al. (2014)
85	glycine	C_2_H_5_NO_2_	Et_2_O	Aerial part	Liu G. C. et al. (2014)
86	alanine	C_3_H_7_NO_2_	Et_2_O	Aerial part	Liu G. C. et al. (2014)
87	cystine	C_6_H_12_N_2_O_3_S_2_	Et_2_O	Aerial part	Liu G. C. et al. (2014)
88	valine	C_5_H_11_NO_2_	Et_2_O	Aerial part	Liu G. C. et al. (2014)
89	dl-methionine	C_5_H_11_NO_2_S	Et_2_O	Aerial part	Liu G. C. et al. (2014)
90	l-isoleucine	C_6_H_13_NO_2_	Et_2_O	Aerial part	Liu G. C. et al. (2014)
91	leucine	C_6_H_13_NO_2_	Et_2_O	Aerial part	Liu G. C. et al. (2014)
92	tyrosine	C_9_H_11_NO_3_	Et_2_O	Aerial part	Liu G. C. et al. (2014)
93	phenylalanine	C_9_H_11_NO_2_	Et_2_O	Aerial part	Liu G. C. et al. (2014)
94	histidine	C_6_H_9_N_3_O_2_	Et_2_O	Aerial part	Liu G. C. et al. (2014)
95	proline	C_5_H_9_NO_2_	Et_2_O	Aerial part	Liu G. C. et al. (2014)
96	lysine	C_6_H_14_N_2_O_2_	Et_2_O	Aerial part	Liu G. C. et al. (2014)
97	arginine	C_6_H_14_N_4_O_2_	Et_2_O	Aerial part	Liu G. C. et al. (2014)
102	**Saccharides and glycosides**				
98	(+)-tortoside A	C_29_H_38_O_12_	EtOAc	Aerial part	Ma et al. (2018b)
99	(-)-tortoside A	C_29_H_38_O_12_	EtOH	Aerial part	Zhou et al. (2017)
100	(3*S*,5*R*,6*R*,7*E*,9*S*)-megastigman-7-ene-3,5,6,9-tetrol-3-*O*-*β*-d-glucopyranoside	C_19_H_34_O_9_	BuOH	Stem	Ouyang et al. (2018)
101	(3*S*,5*R*,6*R*,7*E*,9*S*)-megastigman-7-ene-3,5,6,9-tetrol-9-*O*-*β*-d-glucopyranoside	C_19_H_34_O_9_	BuOH	Stem	Ouyang et al. (2018)
102	(1*R*)-4-[(3*R*)-3-hydroxybutyl]-3,5,5-trimethylcyclohex-3-enyl-*O*-*β*-d-glucopyranoside	C_20_H_36_O_6_	BuOH	Stem	Ouyang et al. (2018)
103	(1*R*)-3-[(4*R*)-4-hydroxybutyl]-2,6,6-trimethylcyclohex-1-methy-propyl-*O*-*β*-d-glucopyranoside	C_19_H_34_O_7_	BuOH	Stem	Ouyang et al. (2018)
104	pinoresinol-4-*O*-*β*-d-glucopyranoside	C_28_H_36_O_11_	BuOH	Stem	Ouyang et al. (2018)
105	syringaresionl-4-*O*-*β*-d-glucopyranoside	C_30_H_40_O_13_	BuOH	Stem	Ouyang et al. (2018)
106	2-(2-hydroxyphenyl)ethanol-*O*-*β*-d-glucopyranoside	C_14_H_20_O_7_	BuOH	Stem	Ouyang et al. (2018)
107	4,6-dihydroxy-2-*O*-*β*-d-glucopyranosyl acetophenone	C_15_H_20_O_9_	BuOH	Stem	Ouyang et al. (2018)
108	methyl-*α*-d-fructofuranoside	C_7_H_14_O_6_	BuOH	Stem	Ouyang et al. (2018)
109	D-Glu-1*α*→2*β*-D-Fru	C_12_H_22_O_11_	BuOH	Secretory	Cheng et al. (2014)
110	D-Glu-1*β*→6-D-Glu-1*α*→2*β*-D-Fru	C_18_H_32_O_16_	BuOH	Secretory	Cheng et al. (2014)
111	**Volatile oils**				
112	isopentane	C_5_H_12_	Aqueous	Secretory	Cheng (2010)
113	pentane	C_5_H_12_	Aqueous	Secretory	Cheng (2010)
114	2,2-dimethylbutane	C_6_H_14_	Aqueous	Secretory	Cheng (2010)
115	2,3-dimethylbutane	C_6_H_14_	Aqueous	Secretory	Cheng (2010)
116	methylcyclopentane	C_6_H_12_	Aqueous	Secretory	Cheng (2010)
117	5-acetyldihydro-2(3*H*)-furanone	C_6_H_8_O_3_	Aqueous	Secretory	Cheng (2010)
118	3-hexanone	C_6_H_12_	Aqueous	Secretory	Cheng (2010)
119	2-ethyl-1-dodecene	C_14_H_28_	Aqueous	Secretory	Cheng (2010)
120	heptane	C_7_H_16_	Aqueous	Secretory	Cheng (2010)
121	hexamethylethane	C_8_H_18_	Aqueous	Secretory	Cheng (2010)
122	methylcyclohexane	C_7_H_14_	Aqueous	Secretory	Cheng (2010)
123	3-methylheptane	C_8_H_18_	Aqueous	Secretory	Cheng (2010)
124	ethylcyclopentane	C_7_H_14_	Aqueous	Secretory	Cheng (2010)
125	1,2,3-trimethyl-cyclopentane	C_8_H_16_	Aqueous	Secretory	Cheng (2010)
126	2,3-dimethylhexane	C_8_H_18_	Aqueous	Secretory	Cheng (2010)
127	2-methylheptane	C_8_H_18_	Aqueous	Secretory	Cheng (2010)
128	octane	C_8_H_18_	Aqueous	Secretory	Cheng (2010)
129	hexanal	C_6_H_12_O	Aqueous	Secretory	Cheng (2010)
130	furfural	C_5_H_4_O_2_	Aqueous	Secretory	Cheng (2010)
131	1,2,5,5-tetramethyl-1,3-cyclopentadiene	C_9_H_14_	Aqueous	Secretory	Cheng (2010)
132	(*E*)-2-hexenal	C_6_H_10_O	Aqueous	Secretory	Cheng (2010)
133	butylacetone	C_7_H_14_O	Aqueous	Secretory	Cheng (2010)
134	heptanal	C_7_H_14_O	Aqueous	Secretory	Cheng (2010)
135	2,7-dimethyloxepine	C_8_H_10_O	Aqueous	Secretory	Cheng (2010)
136	benzaldehyde	C_7_H_6_O	Aqueous	Secretory	Cheng (2010)
137	3-hydroxy-1-octene	C_8_H_16_O	Aqueous	Secretory	Cheng (2010)
138	2,3-octanedione	C_8_H_14_O_2_	Aqueous	Secretory	Cheng (2010)
139	6-methyl-5-hepten-2-one	C_8_H_14_O	Aqueous	Secretory	Cheng (2010)
140	2-pentylfuran	C9H_14_O	Aqueous	Secretory	Cheng (2010)
141	2,4-heptadienal	C_7_H_10_O	Aqueous	Secretory	Cheng (2010)
142	2-cyclohexen-1-one	C_6_H_8_O	Aqueous	Secretory	Cheng (2010)
143	*o*-cymene	C_10_H_14_	Aqueous	Secretory	Cheng (2010)
144	eucalyptol	C_10_H_18_O	Aqueous	Secretory	Cheng (2010)
145	3,7-dimethyl-(*Z*)-2,6-octadienal	C_10_H_16_O	Aqueous	Secretory	Cheng (2010)
146	linalool	C_10_H_18_O	Aqueous	Secretory	Cheng (2010)
147	nonanal	C_9_H_18_O	Aqueous	Secretory	Cheng (2010)
148	6-methyl-3,5-heptadien-2-one	C_8_H_12_O	Aqueous	Secretory	Cheng (2010)
149	thujone	C_10_H_16_O	Aqueous	Secretory	Cheng (2010)
150	camphor	C_10_H_16_O	Aqueous	Secretory	Cheng (2010)
151	4-(5-methyl-2-furyl)-2-butanone	C_9_H_12_O_2_	Aqueous	Secretory	Cheng (2010)
152	4a-methyl-1,2,3,4,4a,5,8,8a-octahydronaphthalene	C_11_H_18_	Aqueous	Secretory	Cheng (2010)
153	2-methyl-3-phenylpropanal	C_10_H_12_O	Aqueous	Secretory	Cheng (2010)
154	vitispirane	C_13_H_20_O	Aqueous	Secretory	Cheng (2010)
155	theaspirane	C_13_H_22_O	Aqueous	Secretory	Cheng (2010)
156	*β*-damascenone	C_13_H_18_O	Aqueous	Secretory	Cheng (2010)
157	tetradecane	C_14_H_30_	Aqueous	Secretory	Cheng (2010)
158	(*E*)-geranylacetone	C_13_H_22_O	Aqueous	Secretory	Cheng (2010)
159	(*E*)-*β*-ionone	C_13_H_20_O	Aqueous	Secretory	Cheng (2010)
160	pentadecane	C_15_H_32_	Aqueous	Secretory	Cheng (2010)
161	aromadendrene vi	C_15_H_24_	Aqueous	Secretory	Cheng (2010)
162	1,6-dioxacyclododecane-7,12-dione	C_10_H_16_O_4_	Aqueous	Secretory	Cheng (2010)
163	hexadecane	C_16_H_34_	Aqueous	Secretory	Cheng (2010)
164	6,10,14-trimethyl-2-pentadecanone	C_18_H_36_O	Aqueous	Secretory	Cheng (2010)
165	farnesylacetone	C_18_H_30_O	Aqueous	Secretory	Cheng (2010)
166	methyl linoleate	C_19_H_34_O_2_	Aqueous	Secretory	Cheng (2010)
167	ethyl oleate	C_20_H_38_O_2_	Aqueous	Secretory	Cheng (2010)
168	1,3,3,4-tetramethylcyclopentene	C_9_H_16_	BuOH	Aerial part	Ma et al. (2018b)
169	heptacosan-1-ol	C_27_H_56_O	EtOAc	Flowers	Guo et al. (2016)
170	**Other constituents**				
171	pinoresinol	C_20_H_22_O_6_	BuOH	Stem	Ouyang et al. (2018)
172	syringaresinol	C_22_H_26_O_8_	EtOH	Aerial part	Zhou et al. (2017)
173	bombasinol A	C_21_H_24_O_6_	EtOH	Aerial part	Zhou et al. (2017)
174	blumenol A	C_13_H_20_O_3_	EtOH	Aerial part	Zhou et al. (2017)
175	abscisic acid	C_15_H_20_O_4_	EtOH	Aerial part	Zhou et al. (2017)
176	niacin	C_6_H_5_NO_2_	BuOH	Stem	Ouyang et al. (2018)
177	dibutyl phthalate	C_16_H_22_O_4_	BuOH	Flowers	Muratova et al. (2019)
178	stigmasterol	C_29_H_48_O	PET	Aerial part	Su et al. (2008)
179	*β*-sitosterol	C_29_H_50_O	PET	Aerial part	Su et al. (2008)
180	12-ene-ursulanol	C_30_H_50_O	EtOAc	Secretory	Cheng et al. (2014)

### Flavonoids

The Fabaceae family is rich in flavonoids, which have anti-inflammatory, antibacterial, and antitumor activity (Wang S. et al., 2019). Flavonoids have been the focus of attention in the field of drug research and development owing to their multiple biological activities and complex mechanisms (Qi and Dong, 2020). So far, over 53 flavonoids (**1–53**) have been reported in *A. sparsifolia*, including 30 flavones, 11 isoflavones, five catechins, five proanthocyanidins, and two flavanones (Guo et al., 2016; Ouyang et al., 2018; Guo et al., 2020). There are 26 flavonoid glycosides containing glucose (**5–6**, **8–9**, **15**, **20**, **28**), galactose (**2**, **46**, **53**), arabinose (**16**, **19**), rhamnose (**23**), robinobiose (**10**, **19**), rutinose (**4, 11, 13–14**, **24**), sophorose (**17**), neohesperidose (**43**), and trisaccharides (**39–41**) (Malik et al., 1997; Su et al., 2008; Zhou et al., 2017). The parent structures of the flavonoids in *A. sparsifolia* are flavones, isoflavones, and catechins, all of which have phenolic hydroxyl substituents. Butin (**1**), the main active monomer in *A. sparsifolia*, can significantly inhibit the proliferation and migration of human cervical cancer Hela cells, human colon cancer HT-29 cells, human liver cancer HepG2 cells, human gastric cancer BGC823 cells, and human oral epidermoid carcinoma kB cells *in vitro* and was found to have synergistic anti-tumor effects in combination with 5-fluorouracil (Ma et al., 2015; Ma et al., 2018). Isorhamnetin-3-*O*-glucoside (**2**), isorhamnetin (**3**), and isorhamnetin-3-*O*-rutinoside (**4**) are the most abundant flavonoids and are often considered important indicators for the quality control of this plant (Xu et al., 2012; Sanawar et al., 2014). Moreover, differences in the total flavonoid content of different parts of the plant have also been reported, with the fruit and aerial parts having the highest content of total flavonoids, suggesting that different medicinal parts can be selected based on clinical use to maximize the utility of this plant (Chen et al., 2014).

### Alkaloids

Alkaloids are important chemical compounds and a good research area for drug discovery. Numerous alkaloids screened from medicinal plants are known to exert antiproliferative and anticancer effects in several cancers both *in vitro* and *in vivo* (Mondal et al., 2019). To date, seven alkaloids have been isolated from this plant, including two organic amines (**59**, **63)**, six pyrrolidines (**54, 57–58**, **60–62**), and three acylamides (**55–56**, **64**) (Cheng, 2010; Zou et al., 2012; Liu G. C. et al., 2014; Zhou et al., 2017). Alhagifoline A (**54**) was the first novel compound isolated from the dried aerial parts of this plant; however, pharmacological studies related to its activity are lacking.

### Phenols, Phenolic Acids, and Amino Acids

Thirty-three organic acids including phenols (**65–66**, **69–71**, **78–79**), phenolic acids (**67–68**, **72–77**, **80**), and amino acids (**81–97**) have been identified from *A. sparsifolia* (Liu G. C. et al., 2014; Cheng et al., 2014; Guo et al., 2016; Zhou et al., 2017; Ma et al., 2018b; Muratova et al., 2019). The substituents of these compounds include hydroxyl, carboxyl, methoxy, amino, and carbonyl groups. Seventeen amino acids have been isolated from the alkaline essential oils of this plant and were reported as the main components of drought resistance in this desert plant (Liu G. C. et al., 2014). The compounds 3-methoxy-4-vinylphenol (**65**) and 3,4-dihydroxybenzaldehyde (**66**) have been shown to inhibit tumor cell proliferation in the concentration range of 25–87 μM. Moreover, owing to their selectivity, these compounds may be used in the development of antitumor drugs in the future (Ma et al., 2018).

### Saccharides and Glycosides

**FIGURE 9:**
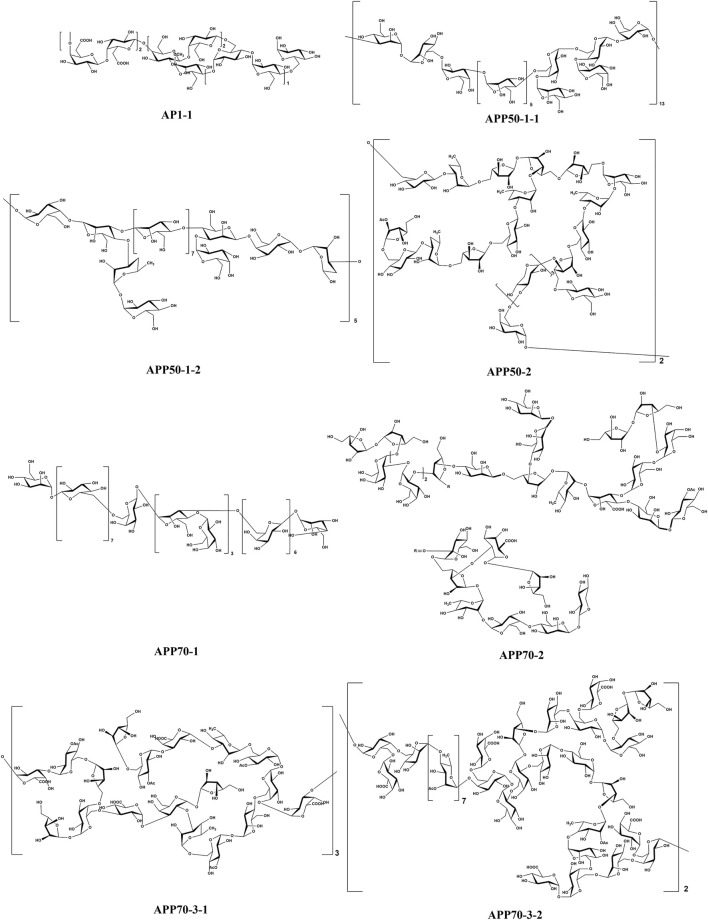
Chemical structures of polysaccharides.

The stems of *A. sparsifolia* are rich in polysaccharides; thus, the isolation and purification of polysaccharides have been the emphasis of research with respect to the chemical constituents of this plant. The 19 isolated polysaccharides (AP1-1, AP1-2, AP1-3, AP1-4, AP1-5, AP2-1, AP2-2, AP2-3, AP2-4, AP2-5, SAP-1, SAP-1, SAP-3, APP50-1-1, APP50-1-2, APP50-2, APP70-1, APP70-2, APP70-3-1, APP70-3-2) contain different amounts of Rha, Ara, Xyl, Man, Glc, Gal, GlcA, and GalA (Chang et al., 2016; Jian et al., 2012; Jian et al., 2014; Li, 2020; Ma et al., 2018; Zheng, 2016; m). The possible chemical structures of seven of these polysaccharides (AP1-1, APP50-1-1, APP50-1-2, APP50-2, APP70-1, APP70-2, APP70-3-1, APP70-3-2) are summarized in Figure 9. The crude polysaccharide extract of *A. sparsifolia* and its monomeric components have significant antioxidant activity and can effectively scavenge free radicals *in vivo*. The higher the molecular weight, the stronger is the scavenging effect (Zhao et al., 2015; Ma et al., 2018). A study has reported the potential hypoglycemic effect of the crude polysaccharide of *A. sparsifolia* and that different doses can prevent the increase in fasting blood glucose levels in diabetic mice. The hypoglycemic mechanism may not be related to oxidative stress capacity (Zhao, 2016). In addition, two oligosaccharides (**103–104**) and 11 oxygen-containing glycosides (**97–107**) have been identified from *A. sparsifolia*, including alcoholic glycosides (**99–102**, **105**, **107**) and phenolic glycosides (**97**–**98**, **103–104**, **106**).

### Volatile Oils

To date, 58 volatile oils (**111–168**) from the secretions of *A. sparsifolia* have been separated and characterized using nuclear magnetic resonance (NMR) and gas chromatography-mass spectrometry (GC-MS) (Cheng, 2010; Guo et al., 2016; Ma et al., 2018b). The isolated compounds are mainly composed of small-molecular lipophilic compounds including monoterpenes (**142–145**, **148–149**), a sesquiterpene (**160**), aliphatic hydrocarbons (**111–114**, **118–120**, **122**, **125–127**, **156**, **159**, **162**), alicyclic hydrocarbons (**115**, **121**, **123–124**, **130**, **151**, **167**), ketones (**117**, **128–129**, **131**–**133**, **135**, **137–138**, **140–141**, **144**, **146–147**, **150**, **152**, **155**, **157–158**, **163–164**), alcohols (**136**, **168**), ethers (**134**, **139**, **153–154**), and esters (**116**, **161**, **165–166**). The structural skeleton of the components consists of five-membered, six-membered, oxygen-containing, and other irregular ring structures. The discovery of several essential oils from *A. sparsifolia* has significantly enriched its chemical database.

### Other Constituents

A few lignans (**169–171**), sterols (**176–177**), triterpenes (**178**), and other compounds with irregular chemical structures (**172–175**) have been isolated from *A. sparsifolia*. There is no common structural skeleton among these components. Compounds (**169**–**173**) were reported to have dose-dependent anti-neuroinflammatory effects.

## Pharmacology

**FIGURE 10:**
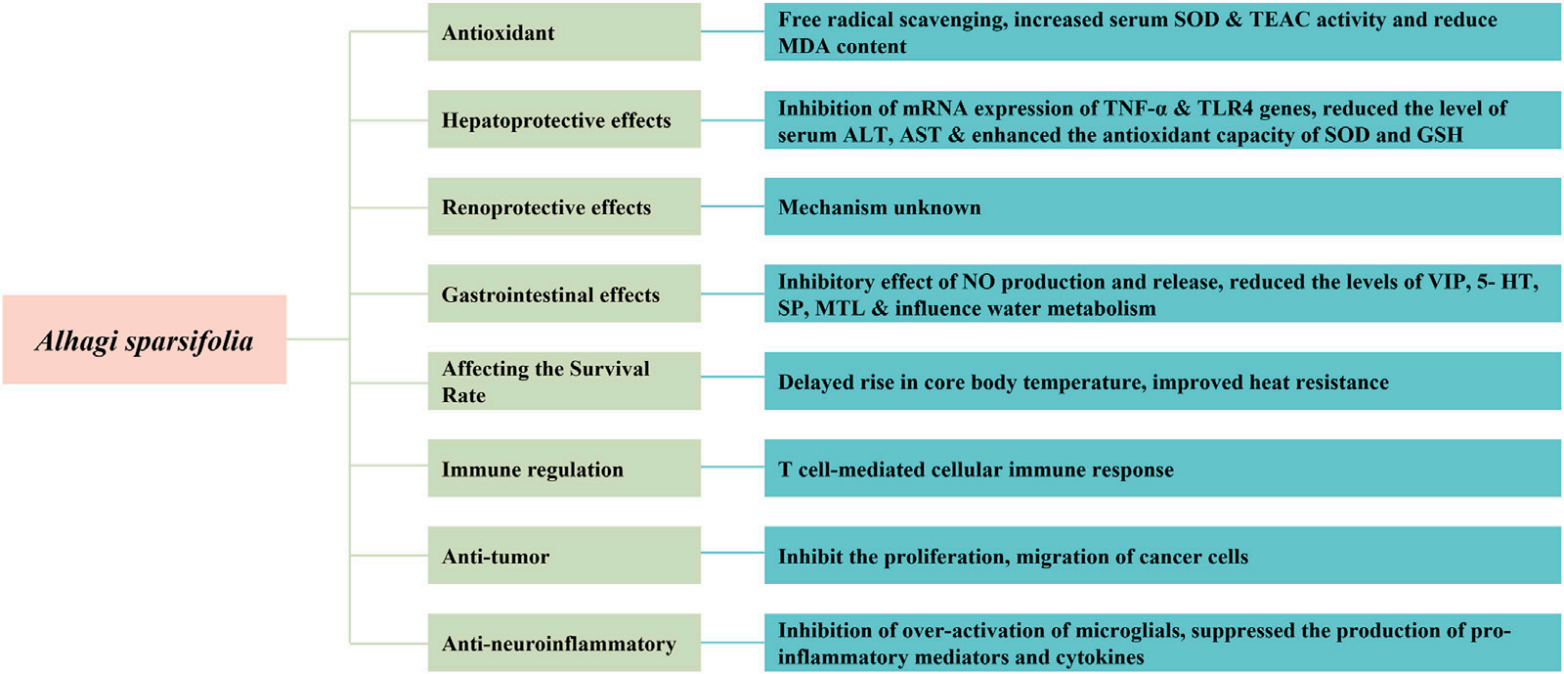
Possible mechanisms for pharmacological activit.

Pharmacological studies have indicated that *A. sparsifolia* has several pharmacological effects, including antioxidant, hepatoprotective, and renoprotective effects. Moreover, it affects the survival rate of rats in a dry and hot environment and plays a role in immune regulation and has antitumor and anti-neuroinflammatory effects. The pharmacological effects of *A. sparsifolia* and its monomers are summarized in Table 3 and their possible potential pharmacological mechanisms are summarized in Figure 10.

**TABLE 3:** Modern Pharmacological studies of *A. sparsifolia*.

Effect	Model	Part of plant/Extracts or compound	Positive control	Formulation/dosage	Result	References
Antioxidant	SOD, MDA, TEAC	Stem-branch/Curde polysaccharide	Lentinan (630 mg/kg) showed similar *in vivo* antioxidant activity to the extract	*in vivo*: 50, 100, 200 mg/kg	Increasing SOD TEAC leveals, decreasing MDA levels	Liu et al. (2021)
Hepatoprotective effects	APAP-induced acute liver injury mice	Secretory/Aqueous	Silibinin (300 mg/kg) significantly inhibits ALT, AST activity and alleviates liver lesions caused by APAP	*in vivo*: 150, 300, 600 mg/kg	Inhibiting the release of ALT and AST caused by APAP overdose and alleviating APAP-induced liver injury and hepatocyte necrosis	Aili et al. (2017)
	Alcoholic-induced acute liver injury mice	Secretory/Aqueous	Silibinin has a protective effect in mice with alcoholic liver disease	*in vivo*: 150, 300, 600 mg/kg	promoting alcohol metabolism, reducing the expression levels of TNF-α and TLR4 mRNA, promoting liver tissue repair and hepatocyte regeneration	Kuerbanjiang et al. (2017)
Renoprotective effects	Gentamicin-induced subacute renal injury mice	Secretory/Aqueous	—	*in vivo*: 350 mg/kg	significant protective effect on renal injury caused by 125 and 80 mg/kg GM, but not on renal injury caused by 100 mg/kg GM	Mikeremu et al. (2013)
	HgCl_2_- induced subacute renal injury in mice	Secretory/Aqueous	—	*in vivo*: 150, 300, 750 mg/kg	Best renal protection at 150 mg/ml	Wumaierjiang et al. (2014)
Gastrointestinal effects	Atropine inhibition and bethanechol chloride promote small bowel motility	Secretory/Aqueous	—	*in vivo*: 750, 1,500, 3,000 mg/kg	Gastrointestinal motility is stimulated and inhibited by excited gastrointestinal motility	Mikeremu et al. (2017)
	Diarrheal irritable bowel syndrome rats	Aerial part/ethanol	TrimebutineMaleate (60 mg/kg) improves diarrhoeal irritable bowel syndrome	*in vivo*: 200, 400 mg/kg	The fecal moisture content, the AWR score and the level of 5- HT, SP, MTL in the high and low dose groups were significantly decreased, and the number of fecal grains increased significantly	Liu et al. (2019b)
	Diarrheal irritable bowel syndrome rats	Aerial part/ethanol	TrimebutineMaleate (60 mg/kg) improves diarrhoeal irritable bowel syndrome	*in vivo*: 200, 400 mg/kg	At the concentration of 400 mg/kg, the extract significantly reduced wall electrical activity and increased *NO* levels	Ma et al. (2018c)
Affecting the survival rate	dry and hot environment rat	Aerial part/ethanol	—	*in vivo*: 100, 330, 1,000 mg/kg	Delaying the rise in core body temperature to improve heat tolerance in dry heat tolerance in rats in a dry heat environment	Dong et al. (2019)
Immune regulation	RAW264.7 cells in mice	Secretory/Aqueous	Lipopolysaccharide (1,000 μg/ml) promote macrophage proliferation but are less active than extract (100, 200 μg/ml)	*in vitro*: 12.5, 25, 50, 100, 200 μg/ml	Promoting macrophage proliferation and having a positive regulatory effect on immune activity	Han et al. (2017)
	CY and DNCB induced delayed type hypersensitivity of immunosuppression mice	Aerial part/BuOH	—	*in vivo*: 100, 200, 400 mg/kg	Enhancing the swollen degree of auricle, resisting the atrophy of spleen and thymus and increasing the index of immune organs	Ma et al. (2017)
Anti-tumor	BGC-82, Eca-10, HT-29 and HepG2 cancer cells	Aerial part/ethanol	The IC_50_ values of Cisplatin for BGC-823, Eca-109, HT-29 and HepG2 cells were 0.019, 0.204, 0.0858, 0.0392	*in vitro*: 10, 5, 2.5, 1.25, 0.625, 0.156, 0.039 mg/ml	The IC_50_ values of BGC-823, Eca-109, HT-29 and HepG2 cells were 1.62, 1.32, 1.55 and 1.45 mg/ml respectively	Ma et al. (2015)
	CT26 colon cancer mice		Inhibition rate of 98% in CT26 colon cancer mice by cyclophosphamide (50 mg/kg)	*in vivo:* 100, 200, 1,000 mg/kg	The inhibition rate was 24.8% in the high dose group, 0.06% in the medium dose group and -0.05% in the low dose group *in vivo*	
	Hela, Ht-29, HepG2, BGC823 and KB tumour cells	Aerial part/EtOAc, BuOH, Compound **11**, **65**, **66**, **98** and **167**	—	*in vitro*: 200, 100, 50, 25, 12.5, 6.25 μg/ml	inhibiting the proliferation and migration of Hela, Ht-29, HepG2, BGC823 and KB tumour cells in a dose-dependent manner	Ma et al. (2018b)
Anti-neuroinflammatory	LPS-induced N9 cells	Aerial part/ethanol	The IC_50_ values of minocycline for LPS-induced N9 cells was 19.89	*in vitro:/*	Compound **3**, **4**, **32**, **37**, **170**, **171**, **54**, and **167** showed much stronger anti-neuroinflammatory effects than minocycline	Zhou et al. (2017)

### Antioxidant

*A. sparsifolia* supposedly exhibit the mostpotent antioxidant activities determined by TEAC assays. Phytochemical studies have shown that *A. sparsifolia* is rich in polysaccharide components, which are closely associated with antioxidant effect (Ma et al., 2018). This indicates that the polysaccharides from *A. sparsifolia* may be a rich source of natural antioxidants that may help prevent and treat diseases related to oxidative stress. Liu et al. (2021) found that the aqueous extract of *A. sparsifolia* stem and branch (ASSBP) demonstrated dose-dependent moderate antioxidant activity when assayed against TEAC, with serum TEAC levels of mice in the low dose group (50 mg/kg) and medium dose group (100 mg/kg) comparable to the positive control group of Lentinan (630 mg/kg) and slightly higher levels in the high dose group (200 mg/kg). The results showed that ASSBP had certain antioxidant ability and enhanced immune activity in both normal and d-galactose-induced aging mice, increased spleen and thymus indices in normal mice, increased serum SOD activity and decreased MDA content in normal mice. It is suggested that its anti-aging effect may be exerted through anti-lipid peroxidation, increasing SOD activity and decreasing MDA content.

### Hepatoprotective Effects

In a study, the aqueous extracts of Tarangabin at the doses of 150, 300, 600 mg/kg had significant hepatoprotective effects at different concentrations in mice with liver injury induced by N-acetyl-*para*-aminophenol (APAP). It showed a significant decrease in the level of serum alanine aminotransferase (ALT) and serum aspartate aminotransferase (AST). The results of content determination showed that the polysaccharide content of the extract was as high as 69.2%, thus it is supposed that the polysaccharides have a controlling effect on APAP-induced liver injury (Aili et al., 2017). The hepatoprotective mechanism of Tarangabin has been partly attributed to the reduction of oxidative stress and inhibition of the expression of cytochrome P450 2E1 (CYP2E1) (Aili et al., 2018). In alcoholic liver disease (ALD), Tarangabin regulates the expression levels of tumor necrosis factor (TNF)-*α* and toll-like receptor 4 (TLR4) mRNA by acting on the lipopolysaccharide-TLR (LPS-TLR) signaling pathway, thereby improving the severity of ALD. Moreover, this plant is known to bring about the effects of reducing the gene expression of TLR4, inhibiting the release of TNF-*α*, preventing further signal transmission, reducing the efficiency of endotoxin signal transduction, and decreasing the production of inflammatory factors, thereby preventing hepatocyte damage. Tarangabin promotes liver tissue repair and hepatocyte regeneration and protects from liver injury (Kuerbanjiang et al., 2017; Wang X. L. et al., 2019).

In addition, *A. sparsifolia* significantly alleviated alcohol-induced liver injury by reducing serum ALT and AST, inhibiting MDA and hydrogen peroxide (H_2_O_2_), and increasing SOD and glutathione (GSH) level in the liver (*p* < 0.05). Additionally, treatment with *A. sparsifolia* was found to significantly reduce the expression of TNF-α and TLR4 mRNA in the brain of mice, thus accelerating alcohol metabolism and reducing oxidative stress by downregulating CYP2E1 expression (*p* < 0.05), which has a protective role in ALD (Kuerbanjiang et al.. 2018).

### Renoprotective Effects

Tarangabin supposedly protected against high-dose gentamicin (GM)-induced acute kidney injury in mice. Although the efficacy is attributed to the organic components and Zn, Fe, Cu, Co, Ni, Mn, and K content of Tarangabin, the specific mechanism of action needs to be further elucidated (Mikeremu et al., 2013). Wumaierjiang et al. (2014) found that Tarangabin had a certain protective effect in acute renal failure caused by mercuric chloride, and the best effect was achieved when the concentration of Tarangabin was 15% (Ma et al., 2018).

### Gastrointestinal Effects

*A. sparsifolia* and Tarangabin have a dual regulatory effect on the small intestinal motility in mice. The aqueous extract of *A. sparsifolia* and Tarangabin could inhibit intestinal propulsion induced by the M-choline receptor blocker, atropine sulfate, and alleviate hyperactivity of small intestinal propulsion induced by the M-choline receptor stimulant, carbamyl-B-methylcholine chloride, in a dose-dependent manner. The dual regulation effect of *A. sparsifolia* and Tarangabin may be attributed to the high taurine content in the plant. It has been reported that taurine has a dual regulatory effect on intracellular Ca^2+^. A certain dose of taurine can enhance intestinal smooth muscle contraction, but the effects of different concentrations of taurine on smooth muscle are different. Low and medium doses of taurine enhance smooth muscle contraction, whereas high doses of taurine have the opposite effect (Mikeremu et al., 2017).

In a particular study, male Sprague-Dawley (SD) rats were subjected to restraint stress and fed a high-lactose diet to establish a diarrhea-predominant model of irritable bowel syndrome (IBS-D), which was further used to evaluate the regulatory effect of *A. sparsifolia* extract in IBS-D. The extract was found to significantly reduce the fecal water content and abdominal wall myoelectric activity, and significantly increase the volume threshold of abdominal contractile reflex and serum nitric oxide (NO) levels. Additionally, *A. sparsifolia* extract was found to improve visceral hypersensitivity and decrease the myoelectric activity of the abdominal wall in IBS rats, which may be related to the decrease in the secretion and release of NO (Wei et al., 2016; Ma et al., 2018c).

In a recent study, Liu et al. (2019b) have shown that in IBS-D rats, *A. sparsifolia* extract can decrease the abnormally elevated levels of the gastrointestinal hormones, 5-hydroxytryptamine (5-HT), substance P (SP), and motilin (MTL); regulate water metabolism to inhibit gastrointestinal motility and delay gastric emptying; and improve abdominal distension, abdominal pain, and diarrhea, which may be the mechanism of this botanical drug in the treatment of this condition. However, the tension of the intestinal smooth muscle and related intestinal electrolyte levels were not observed in this study, and the vasoactive intestinal peptide (VIP) levels following the administration of a high dose of *A. sparsifolia* extract was not higher than that observed after administration of a low dose of the extract. The antagonism between various components in the extract or the effect of some components in the extract as autoinducers may be the likely causes of this abnormal phenomenon.

### Affecting the Survival Rate of Rats in a Dry, Hot Environment

The incidence of summer heat strokes and heat radiation in dry and hot desert environments is increasing every year. Dong et al. (2019) found that the desert plant, *A. sparsifolia*, can improve the heat tolerance of rats in a dry and hot environment by delaying the increase in core body temperature, thereby improving their survival rate in dry and arid conditions.

### Immune Regulation

Results from *in vitro* cellular assays suggested that SAP-1, SAP-2, and AP1-1 promoted the proliferation and cellular immunity of splenic lymphocytes and RAW264.7 macrophages. These components could promote the secretion of cytokines IL-1β, IL-6, IL-12, and NF-κB. The mechanism of action was via the MyD88-dependent pathway, which activated the immune response of TLR4 receptors (Han et al., 2017). Results from *in vivo* experiments in mice indicated that the polysaccharide extract could enhance carbon particle scavenging ability, increase serum hemolysin levels in immunosuppressed mice, and also increase serum IL-2 and IL-6 levels in cyclophosphamide-induced immunosuppressed mice (Zhao, 2016; Zhao et al., 2016; Qu, 2019). In addition, *A. sparsifolia* extract was found to significantly increase the number of white blood cells and lymphocytes in peripheral blood, reduce the atrophy of immune organs, improve the ability of lymphocyte transformation in cyclophosphamide-induced immunosuppression, and enhance delayed hypersensitivity in mice (Ma et al., 2017).

### Anti-Tumor

The inhibitory effects of ethanol extracts from aerial part of *A. sparsifolia* on human gastric cancer (BGC-823), human esophageal cancer (Eca-109), human colon cancer (HT-29) and human hepatocellular carcinoma (HepG2) cells were investigated *in vitro* by MTT assay with IC_50_ of 1.62, 1.32, 1.55 and 1.45 mg/ml, respectively. To further clarify its *in vivo* antitumor efficacy, a CT26 mouse model of colon cancer was established. The *in vivo* tumor-inhibition activity was investigated by comparing the tumor-proliferation rate, growth curve, and tumor-inhibition rate in each group of mice. The results showed that only the high dose (1,000 mg/kg, intragastric × 14 qd) led to significant *in vivo* antitumor activity with a tumor-inhibition rate of 24.8%. Compared with that in the negative control group, serum IL-2 levels of mice that received a high dose of the extract increased significantly. Thus, the antitumor mechanism may be related to the increase in IL-2 (Ma et al., 2015).

Two active monomers, butin (**1**) and 3,4-dihydroxybenzaldehyde (**66**), extracted from the n-butanol extract of *A. sparsifolia,* inhibit the proliferation and migration of human cervical cancer (HeLa) cells and exert a strong inhibitory effect *in vitro* with synergistic antitumor effects with 5-FU. However, the related targets and signaling pathways of its antitumor effects are still unclear (Liu et al., 2019a). Using Hela cells as the target cells, the proliferation inhibition ability of each different polar extracts on tumor cells was detected by MTT assay, and butanol and ethyl acetate extracts among them were clearly shown to have certain inhibition effect on the proliferation of tumor cells. Further studies proved that butin (**1**), (+)-tortoside A (**98**), 3-methoxy-4-vinylphenol (**65**), 3,4-dihydroxybenzaldehyde (**66**), and 1,3,3,4-tetramethyl-cyclopentene (**167**) were the active monomers exerting antitumor effects. They also showed good inhibitory effects on HT-29, HepG2, BGC823, and KB tumor cells, exhibiting potential as antitumor agents (Ma et al., 2018b).

### Anti-Neuroinflammatory Effects

Microglia cells are considered to be key innate immune cells in the central nervous system (CNS) and an important contributor to neuroinflammation. However, microglia cells are particularly sensitive to changes in their microenvironment and readily become activated in response to infection or injury. Under activated conditions, they secrete and release a mass of pro-inflammatory cytokines, including tumor necrosis factor-α (TNF-α), interleukin-1β (IL-1β), interferon-γ (IFN-γ), interleukin-6 (IL-6), and free radical mediators such as nitric oxide (NO) and reactive oxygen species (ROS). Accumulation of the pro-inflammatory and neurotoxic factors might aggravate the pathogenesis of neurodegenerative diseases. The LPS-stimulated N9 cells were used to evaluate the anti-neuroinflammatory activity of 33 compounds isolated and identified from the ethanol extracts from aerial part of A. sparsifolia. Among the examined constituents, compounds **1**, **3**, **4**, **15**, **32**, **36**, **37**, **42**, **54**, **65**, **79**, **99**, **167**, **169**, **170**, **171**, **172**, and **173** could considerably inhibit NO production in LPS-induced N9 microglial cells in a dose-independent manner without evident cytotoxicity at the tested concentrations. The effect of 33 compounds on the anti-neuritis activity of N9 cells by MTT assay revealed that flavonoids and lignans exhibited anti-inflammatory effects, whereas their glycosides were not as effective (compound **3** vs **40** [IC_50_ 17.87 vs > 100], **170** vs **105** [IC_50_ 2.68 vs > 100]). In addition, isorhamnetin (**3**) (IC_50_ 17.87 μM), quercetin (**4**) (IC_50_ 10.22 μM), 3′,7-dihydroxyl-4′-methoxylisoflavone (**32**) (IC_50_ 17.43 μM), 3′,7-dihydroxyl-4′,6-dimethoxyl isoflavone (**37**) (IC_50_ 11.21 μM), syringaresinol (**170**) (IC_50_ 2.68 μM), bombasinol A (**171**) (IC_50_ 7.61 μM), aurantiamide (**54**) (IC_50_ 14.91 μM), and 1,3,3,4-tetramethylcyclopentene (**167**) (IC_50_ 2.63 μM) showed much stronger anti-neuroinflammatory effects without obvious cytotoxicity at their effective concentration compared with the positive control, minocycline (IC_50_ 19.89 μM). The mechanism of action may be related to the inhibition of excessive activation of microglia and thus the inhibition of the production of pro-inflammatory mediators and cytokines (Zhou et al., 2017).

### Other Activities

In addition to the above pharmacological effects, the isolated compounds and crude extract of *A. sparsifolia* showed antibacterial, antidiabetic, and growth-promoting effects. The aqueous extract exhibited good antibacterial activity against *Escherichia coli* and *Staphylococcus aureus* and had a minimum inhibitory concentration (MIC) of 62.5 mg/ml (Lei et al., 2004). A gavage of the polysaccharide (at a dose of 200 mg/kg) administered to male mice with hyperglycemia significantly suppressed fasting blood glucose levels (Zheng, 2016). Moreover, the polysaccharides were found to exert growth-promoting and hemoglobin-increasing effects in mice (Hairula et al., 2014).

## Quality Control

Although Uyghur medicines have shown unique efficacy in the treatment of several diseases and gained increasing attention and recognition, there is still a big gap in the industrialization, standardization, and the mode of standardization of Uyghur medicines. First, there is the problem of poor basic research. According to statistics, there are still about 250 Uyghur drugs without defined standards among the 500 commonly used drugs. Some of the drugs for which standards are available have unclear identification of origin, phenomena of synonym or homonym, translation errors, and unverified Latin names. Secondly, the level of quality standards of plant species used in Uyghur medicine species is low, the number of standardized species is small, and the identification specificity is not strong; thus, effective quality control of Uyghur medicine poses a challenge. Thirdly, the scientific clinical research evaluation system including the clinical efficacy evaluation index and research methods of Uyghur medicine has not been established, and evaluation of the clinical efficacy of Uyghur medicine lacks a standardized, objective, and recognized index. There is a lack of in-depth research and exploration of the rationality of the composition of ethnic medicines, the scientific nature of the process, and the active components of drugs. Therefore, it is crucial to establish complete quality standards and suitable extraction methods for the quality control of *A. sparsifolia*.

### Studies on the Quality Standards of *A. sparsifolia*

*A. sparsifolia*, as a common ethnic medicine in Uyghur medicine, has a long history of use. Presently, *A. sparsifolia* is not included in Pharmacopoeia of the People’s Republic of China (ChP). With continuous improvements in modern separation and identification methods, some investigators have used various methods to evaluate the chemical compounds and control the quality of *A. sparsifolia*. For example, Hu (2010) determined the moisture, total ash, leachate, and total flavonoid content of *A. sparsifolia* in Tuokexun County, Xinjiang, by referring to the identification items and standardized methods in the ChP. The specificity of isorhamnetin (**3**), isorhamnetin-3-*O*-glucoside (**17**), and isorhamnetin-3-*O*-rutinoside (**12**) in *A. sparsifolia* was determined using TLC, whereas the levels of the main chemical component, isorhamnetin-3-*O*-rutinoside, were determined using HPLC and found to be 0.1355%, on average. In the same year, Sanawar et al. (2012) conducted a similar study on 12 *A. sparsifolia* samples collected from the Turpan region of Xinjiang. In addition, they focused on describing the plant traits and determining isorhamnetin content using HPLC. The average isorhamnetin content was determined to be 0.14%. To improve the credibility and accuracy of the quality standard, the XinJiang Institute of Chinese Traditional Medica and Ethical Materia Medica conducted a more comprehensive quality standard study on samples collected from five different regions in Xinjiang by random sampling. The findings revealed that the impurities did not exceed 0.5%, moisture content did not exceed 10%, and total ash content did not exceed 12%. Moreover, unique TLC and HPLC methods for rutin were established (Xu et al., 2012). In addition, Chen et al. (2014) determined the levels of total polysaccharides and rutin in eight samples of *A. sparsifolia* using ultraviolet spectrophotometry (UVS) and compared its levels in different parts of the plant. The results showed that the total polysaccharide and rutin content in the fruit and aerial parts were relatively high. In another study, HPLC and similarity evaluations were used to establish the chromatographic fingerprints of 10 *A. sparsifolia* samples collected from different townships in Tuokexun County, Xinjiang (Sanawar et al., 2013), which revealed 11 common peaks in the fingerprints of *A. sparsifolia* obtained from 10 habitats. However, there were differences in the peak heights of the common peaks in *A. sparsifolia* obtained from different habitats, indicating differences in the levels of the primary components of botanical drugs obtained from different sources due to local climate and harvesting time. Guo et al. (2020) were the first to use UPLC-Q-TOF-MS for the qualitative analysis of flavonoids from Tarangabin. Using a web-based database and masslynx 4.1 workstation, 40 compounds were analyzed and identified, of which 22 were reported for the first time. Their study provides a scientific basis for the establishment of a compound database and quality standards. Establishment of quality standards for *A. sparsifolia* will accelerate its entry into the ChP and provide theoretical support and serve as a reference standard for its use in a clinical setting.

### Extraction and Separation Methods

Flavonoids are the main components and active compounds in *A. sparsifolia*; thus, optimizing their extraction is essential for quality control and ensuring efficacy. Su et al. (2009) found that the extract (1 g: 20 ml) of *A. sparsifolia* collected from Tuokexun County purified with 40% ethanol (1.5 h × 3 times) could extract 1.70% of the total flavonoid based on orthogonal experiments. At a temperature of 90°C, its extracts (1 g: 20 ml) were purified with 40% ethanol (1 h × 3 times), and the extraction ratio of total flavonoids was 1.33% when *A. sparsifolia* samples from Urumqi, Xinjiang, were used (Shi et al., 2014). Guo et al. investigated the enrichment ability of AB-8, DM301, and D-101 macroporous resins for total flavonoids in prickly sugars, and finally selected AB-8 resin for the enrichment and purification of total flavonoids from prickly sugars. The ideal extraction process was obtained based on Box-Behnken response-surface optimization, wherein ethanol concentration was 67%, the material:liquid ratio was 1:25, extraction time was 75 min, and extraction temperature was 75°C. The average total flavonoid yield extracted from Tarangabin collected from Hotan, Xinjiang was 0.3889% (Guo et al., 2020).

## Toxicology

It is well known that drugs have dual effects, namely therapeutic effects and adverse reactions. In recent years, Uyghur medicines have been widely used and the incidence of adverse reactions has increased accordingly, thereby raising serious questions regarding their safety in a clinical setting. Toxicity studies on Uyghur drugs will help provide a reference for drugs in the treatment of diseases and ensuring the safe use of drugs. Chronic toxicity studies of different doses (3.0, 1.0, 0.3 g/kg) of the total flavonoid extract from the aerial parts of *A. sparsifolia* in mice show that the routine hematological and biochemical indices after 13 weeks of gavage were not different compared with the control group. Furthermore, no significant differences were found between the control and drug-treated groups, and the isolated organs did not exhibit any pathological changes following drug treatment, indicating that *A. sparsifolia* extracts to be safe over a wide dose range. Similar results were obtained for the total alkaloid extracts from the aerial parts of *A. sparsifolia* (Ma et al., 2014; Zheng et al., 2014). Nevertheless, these findings constitute insufficient evidence to prove the nontoxicity and safety of *A. sparsifolia.* Acute toxicity experiments should be conducted to assess the safety and reliability of this drug when administered at regular doses.

## Future Outlooks

In this review, we have provided a critical analysis of the botany, traditional uses, phytochemistry, pharmacology, quality control, and toxicology of *A. sparsifolia*, which is widely used in the traditional Uyghur system of medicine for the treatment of colds, rheumatic pains, diarrhea, stomach aches, headaches, and toothaches. Modern pharmacological studies have shown the plant components to exert antioxidant, antineuritic, antitumor, immunomodulatory, hepatoprotective, and renoprotective effects. With an improvement in extraction techniques and advancement in pharmacological research, some success has been achieved in determining the chemical composition and pharmacological effects of this plant. However, further studies are warranted for a more thorough understanding. Therefore, we have highlighted and summarized a few topics, which should be investigated further.

First, owing to a lack of basic research in the Uyghur medical system, there exist problems of inaccurate plant nomenclature, leading to misuse and confusion with respect to their use. Kurban and Vonlanthen believe that *Alhagi pesudalhagi*, *Alhagi kirghisorum* Schrenk, *Alhagi maurorum* Medik, and *Alhagi camelorum* Medik all refer to the plant (*A. sparsifolia*) (Kurban et al., 1998; Vonlanthen et al., 2010; Yuan et al., 2012). At present, there is only one species of *A. sparsifolia* in the Flora of China, and it is believed that *A. pseudalhagi*, *A. maurorum* var. Sparsifolium, and *A. camelorum* are synonyms for this plant. However, in the NCBI and TPL databases, *A. sparsifolia* Shap, *A. pseudalhagi*, *A. camelorum* Medik, and *A. kirghisorum* Schrenk belong to different species of the same genus. The literature search revealed that *A. pesudalhagi* and *A. sparsifolia* were used interchangeably in many studies, which also indicates that this review is based on literature that may not be very reliable. Furthermore, the safety and quality evaluation of this plant has been reported in several studies; however, the established evaluation methods are nonstandardized and incomplete due to the technical and methodological limitations at that time. Several researchers have evaluated the quality of *A. sparsifolia* from different origins or different harvesting periods and analyzed the similarities and differences of their chemical composition. However, one or two samples were often used to represent an appellation or even a province, and the reliability of the quality evaluation was greatly diminished by using a certain component (total flavonoids or polysaccharides) or even a single component (isorhamnetin or its glycosides) for quality evaluation. Therefore, standardized medicinal standards should be established to guide the medicinal use and ensure the quality of drug preparations when using this medicinal plant.

**FIGURE 11:**
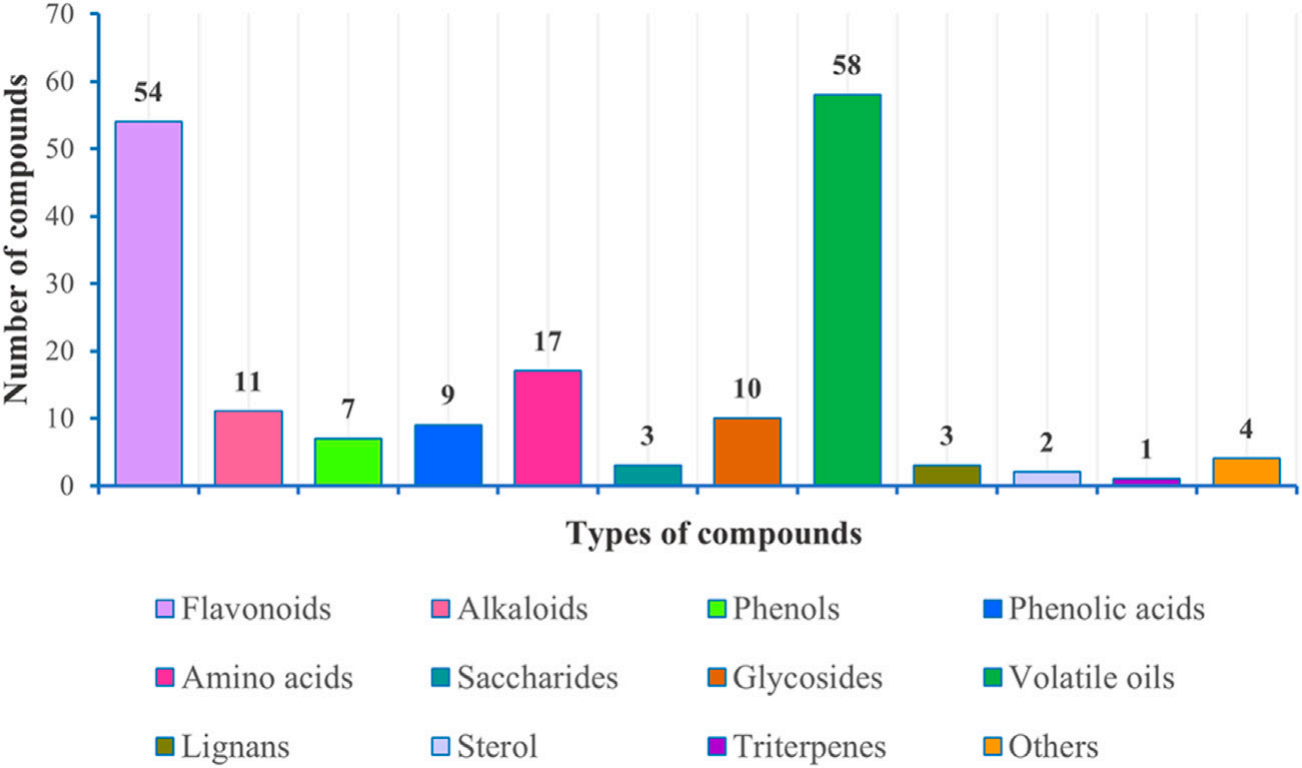
Percentage of compounds in different parts of *A. sparsifolia*.

**FIGURE 12:**
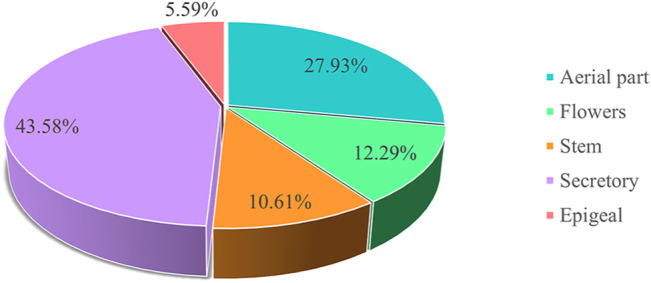
Types and number of compounds isolated from the different part of *A. sparsifolia*.

Second, we determined the extent of research conducted on the chemical composition of different medicinal parts of the plant, and the percentage (%) of active components was calculated based on the number of isolated compounds from extracts of different parts of the plant (Figure 11). To further clarify the main chemical constituents in the plant, the number of different types of compounds was statistically analyzed (Figure 12). By comparing the data, we found that the study of the chemical composition of *A. sparsifolia* was mainly focused on the secretory parts (43.58%), aerial parts (27.93%), flowers (12.29%), stem (10.61%), and epigeal parts (5.59%), with almost no studies on its seeds and roots. Tannins, saponins, and coumarins have been isolated from the seeds and roots of other plants from the same genus, their pharmacological activities have been demonstrated, and their presence has been confirmed using colorimetric assay; however, similar studies for *A. sparsifolia* have not been reported (Cheng, 2010; Srivastava et al., 2014; Muhammad et al., 2015). Therefore, the chemical constituents of the seeds and roots should be studied with the aim of isolating the active compounds and enriching the existing knowledge of the chemical constituents of *A. sparsifolia*. Although a wide range of pharmacological activities of various flavonoids and polysaccharides isolated from *A. sparsifolia* have been reported, research on the pharmacological effects and targets of alkaloids, phenols, and phenolic acids is relatively scarce, which deserve to be further explored. The compounds with the parent nucleus structure of flavone are the most abundant chemical constituents of the plant, among which Butin (**1**) is the material basis for the significant *in vitro* and *in vivo* antitumor effects of the plant. It is necessary and meaningful to further verify whether other compounds with the same parent nucleus structure have the same antitumor activity. Polysaccharides are considered to be biologically active components with antioxidant, hepatoprotective effects, renoprotective effects, regulation of the intestinal and immune regulation. However, polysaccharides are structurally complex and difficult to isolate, and it is challenging and promising to carry out studies on their chemical composition. Furthermore, most studies have focused on the analysis of serum biochemical parameters and cellular expression levels without the in-depth exploration of specific mechanisms and pharmacological targets, severely limiting the possibility of exploring other potential pharmacological activities and developing the active components as new drugs. Therefore, the chemical composition of *A. sparsifolia* should be further elucidated to better understand the pharmacological activity and specificity of each compound.

Third, Numerous studies have shown that *A. sparsifolia* may be a promising candidate for the treatment of cancer and alcoholic liver disease. Flavonoid and phenolic acid active compounds from *A. sparsifolia* exert their antitumor effects mainly through inhibition of proliferation and migration, induction of apoptosis and improvement of immune function. The hepatoprotective mechanism is attributed to the reduction of oxidative stress and inhibition of CYP2E1 expression, and improvement of ALD by acting on the LPS-TLR signaling pathway to regulate the expression levels of TNF-α and TLR4 mRNA. Nevertheless, the relevant targets and signaling pathways for the anti-tumor effects and the specific active compounds for the hepatoprotective effect are still unclear and need further investigation. Polysaccharides are a class of natural macromolecules with various pharmacological activities, and the results have shown that polysaccharides in A. sparsifolia can be used for the prevention and treatment of diseases related to aging and oxidative stress, and are a class of natural antioxidants worth developing. *A. sparsifolia* has also been reported to have antidiabetic, antibacterial, anti-inflammatory, immunomodulatory and gastrointestinal effects, but the studies are not comprehensive enough and its relevant pharmacological properties need to be confirmed by designing additional and more in-depth pharmacological experiments. In addition, *A. sparsifolia* has been used as ethnic medicine by Uyghurs for hundreds of years and numerous empirical prescriptions known for their significant therapeutic effects have emerged. Nevertheless, the relationship between traditionally reported outcomes and modern pharmacological activity has not been thoroughly investigated. Traditionally, the plant has been used in the treatment of pain in various parts of the body owing to its unique efficacy, but modern pharmacological studies have not yet identified any compounds in this plant that are analgesic in nature. *A. sparsifolia* belongs to class II wet-heat drugs in the Uyghur system of medicine, in which drugs exhibiting antihypertensive, anticancer, and antidiabetic effects are included; however, no scientific experiments have been designed to prove these effects. The urinary tract effect, antipyretic effect, musculoskeletal effect and cardiac effect have been already investigated in other plants of the same genus, but so far no similar activity has been reported in this plant and the studies of similar activity are the focus of future research on this plant (Tavassoli, 2020). Chronic toxicity tests reveal the safety and nontoxicity of *A. sparsifolia*; however, this evidence may be insufficient. Therefore, additional studies evaluating the acute toxicity, safety profile, reproductive toxicity, and genotoxicity are warranted to systemically evaluate the toxicity of the extract or its chemical constituents using various animal models. Although the entire plant or its single components have been widely used by Uyghurs, few studies have reported the pharmacokinetic parameters such as the C_max_, T_1/2_, area under the curve, and bioavailability of the monomers or extracts in *in vivo* studies. Therefore, pharmacokinetic studies to obtain relevant parameters for further clinical studies are much needed.

Lastly, other studies have also shown that *A. sparsifolia* exhibits cold resistance, drought resistance, salt tolerance, and wind-sand resistance, and plays an important role in land reclamation and preventing land from being eroded by wind-blown sand (Dong, 2000; Yi, 2020). It is rich in crude protein and crude fat, and these levels can reach 14.2 and 3.5%, respectively, during flowering, making it one of the most nutritious forage grasses in desert areas (Jin, 1994; Wang Y. J. et al., 2019). It is also considered a source of nectar and is known to have several advantages including its widespread distribution, long flowering period, high honey secretion, and high sugar content (Gao, 2005). Moreover, it plays a key role in improving the salinity of soils and enhancing nitrogen cycling in the ecosystem. These findings suggest that *A. sparsifolia* is not only a medicinal plant with potential for the treatment of diseases but is also valuable in environmental protection. However, with environmental and climatic changes, the suitability of the habitat for *A. sparsifolia* is decreasing and the abundance of this species shows a downward trend. Thus, *A. sparsifolia* reserves should be established according to local conditions. On the one hand, the establishment of a plant reserve can protect this rich medicinal resource. More importantly, this step can help effectively curb desertification of the oasis and play a role in improving and stabilizing the ecology of the region.

To summarize, *A. sparsifolia* is a valuable and abundant medicinal resource with promising therapeutic properties and good scope for further exploration. Going forward, more comprehensive studies on the characterization of its active ingredients, determination of its pharmacological mechanisms, and establishment of quality control and toxicity are extremely important to further validate the clinical efficacy and safety of *A. sparsifolia* extracts and isolated bioactive components.

## Glossary

ALD: alcoholic liver disease
IL-2: interleukin-2
ALT: alanine aminotransfease
LPS-TLRs: lipopolysaccharide-toll-like receptors
APAP: *N*-Acetyl-*para*-aminophenol
MDA: malondialdehyde
ASSBP: *A. sparsifolia* stem-branch
MTL: motilin
AST: aspartate transaminase
NO: nitric oxide
BGC-823: human gastric cancer cells
RP-HPLC: reversed-phase high performance liquid chromatogra
**Ca** ^**2+**^: calcium
SD: sprague-dawley
ChP: pharmacopoeia of the people’s republic of china
SOD: superoxide dismutase
CY: cyclophosphamide
SP: substance p
CYP2E1: cytochrome p450 2e1
TEAC: trolox equivalent antioxidant capacity
DNCB: 2,4-dinitrochlorobenzene
TLC: thin-layer chromatography
DPPH: 1,1-diphenyl-2-picrylhydrazyl
TLR4: toll like receptor 4
Eca-109: human esophageal cancer cells
TNF-*α*: tumor necrosis factor-*α*
GM: gentamicin
UVS: ultraviolet spectrophotometry
GSH: glutathione
VIP: vasoactive intestinal peptide
**H**_**2**_ **O**_**2**_: hydrogen peroxide
**•O2** ^**−**^: superoxide anions radical
HepG2: human hepatocellular carcinoma cells
**•OH** ^**−**^: hydroxyl radicals
HPLC: high performance liquid chromatography
5-FU: 5-fluorouracil
HT-29: human colon cancer cells
5-HT: 5-hydroxytryptamine
IBS-D: irritable bowel syndrome model
MIC: minimum inhibitory concentration
**IC**_**50**_: 50% inhibitory concentration
