# Supplementary figures and images for: Traditional Uses, Chemistry, Pharmacology, Toxicology and Quality Control of Alhagi sparsifolia Shap.: A Review
Source: Front Pharmacol. 2021 Oct 14;12:761811. doi: 10.3389/fphar.2021.761811 (PMC8551495; doi:10.3389/fphar.2021.761811)

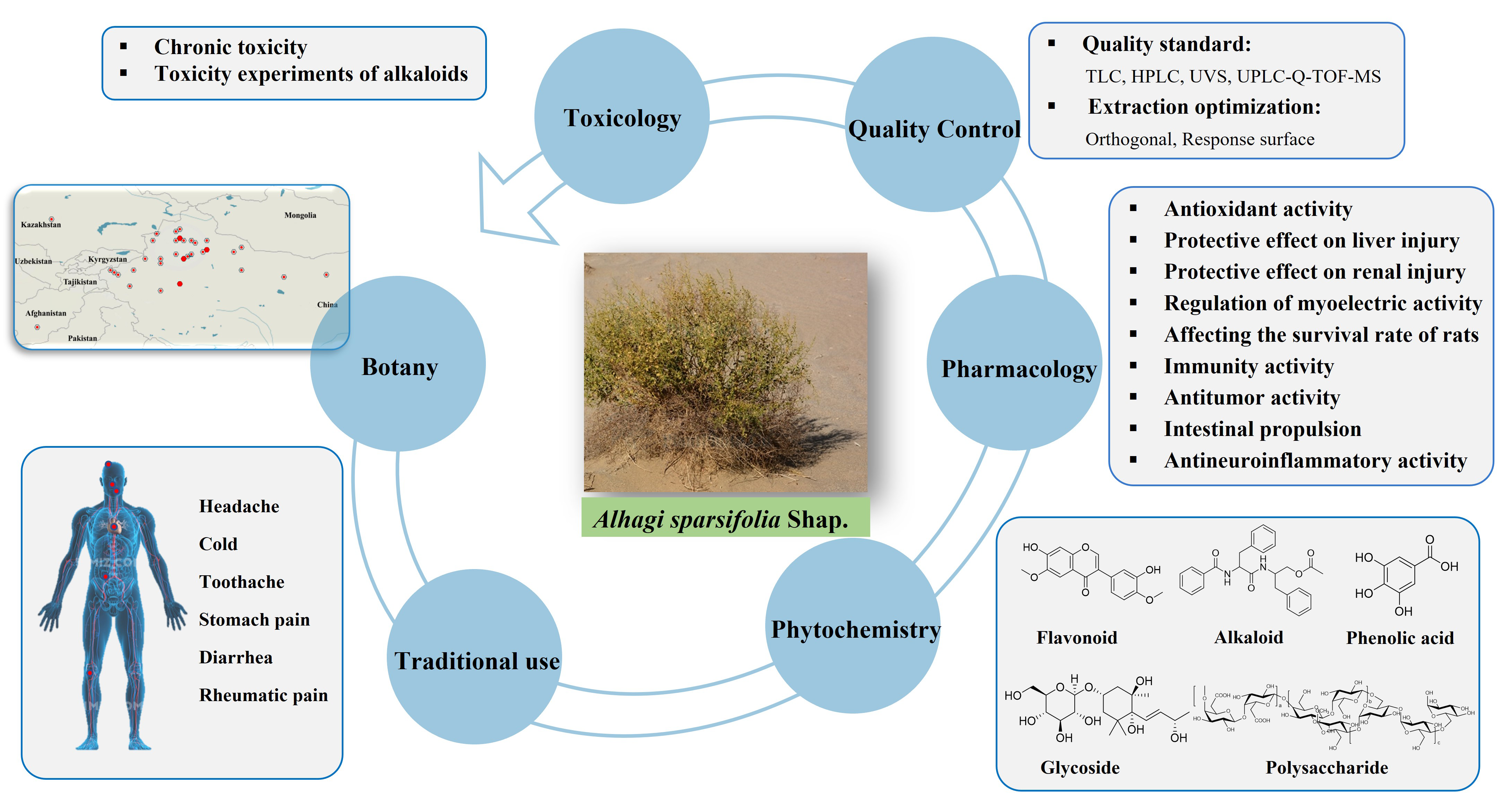

Supplement: Supplementary file 1 [file Image1.TIF]
